# Stress Processes and Resilience Pathways of Sexually and Gender Diverse Communities: A Narrative Review of 15 Years of Evolving Research Priorities and Practices

**DOI:** 10.1002/ajhb.70323

**Published:** 2026-08-27

**Authors:** Robert‐Paul Juster

**Affiliations:** ^1^ Department of Psychiatry and Addiction University of Montreal Montreal Quebec Canada

**Keywords:** allostatic load, cortisol, gender identity, resilience, sexual and gender minority stress, sexual orientation, stress physiology

## Abstract

Sexually and gender diverse (SGD) people in North America have navigated significant sociopolitical shifts that continue to shape their lived experiences. Given the persistent exposure to stigma, SGD communities are disproportionately vulnerable to stress‐related health disparities. While sexual and gender minority stress models highlight the detrimental impact of stigma, research also emphasizes the role of protective factors and resilience pathways in mitigating these effects. This narrative review of my research program over the last 15 years explores stress physiology as a potential mechanism linking stigma to long‐term health and wellness trajectories among SGD populations. Through a selection of key findings and that of my collaborators, I discuss the physiological underpinnings of stress adaptation among SGD people and our evolving research approaches in partnership with the community. Specifically, my work examines allostatic load, a measure of cumulative physiological “wear and tear” resulting from chronic stress. While our initial work focused on the impacts of marginalization, this work increasingly highlights the strengths and adaptive capacities within SGD communities. By bridging stress physiology with resilience frameworks, we aim to inform interventions that promote health equity and well‐being for SGD communities worldwide.

## 1 Introduction

More than half a century ago, *sexually and gender diverse* (SGD) people living in North America partook in two major sociopolitical shifts. In the United States, the 1969 Stonewall riots galvanized the SGD community's civil rights movement. That same year in Canada, the federal government decriminalized homosexuality with Bill C‐150, following Québec's initiative as the first jurisdiction in North America to do so. Since then, SGD communities have fought hard to garner gains (e.g., marriage equality) but have also faced terrible losses (e.g., HIV/AIDS pandemic, rollback of rights). The Covid‐19 crisis and its aftermath has been particularly problematic for SGD people who already faced numerous health and social inequalities pre‐pandemic as compared to *cisgender heterosexual* people.

The *two‐spirit*, *lesbian*, *gay*, *bisexual*, *transgender*, *queer*, *intersex*, and *asexual* (2S/LGBTQIA+) community continues to experience significant health disparities in physical health and mental well‐being (IOM 2011; National Academies of Sciences 2020). Recently, sociopolitical climates have become ever more sexist, racist, homophobic, and especially transphobic in the United States (Dubois, Puckett, et al. 2022; DuBois, SturtzSreetharan, et al. 2022; Puckett et al. 2022) as well as in Canada (Trianon 2025). In writing and revising this manuscript, a second Trump presidency is actively dismantling the rights of American SGD citizens and attempting to render the 2S/LGBTQIA+ community invisible by removing *sexual orientation and gender identity* (SOGI) measures from national surveys and cuts in federal funding. This was also feared in Canada in the expected vote for a conservative government in our last election that instead maintained a liberal leadership. In North America and indeed in many countries around the world, homophobic, transphobic, and fundamentally sexist policies have damaging effects on SGD communities that I fear can have long‐lasting impacts. Integrating and maintaining SOGI measurement to the study of stress experienced by SGD people is an essential endeavor to address health disparities. This is impossible to achieve for invisible populations with unique health and wellness needs.

Beyond stigma, stress, and strain, SGD communities also demonstrate remarkable strength and community resilience. Coping strategies and buffering effects derived from protective processes can help dampen the negative effects of stigma exposure on health and well‐being over time. Indeed, stress processes and resilience pathways are increasingly conceptualized not as static states, but rather as probabilistic processes that vary according to the contexts and the consequences investigated (Cicchetti and Toth 2009; Rutter 1985; Rutter and Sroufe 2000). From this perspective, *resilience* is the dynamic process that promotes positive adaptation among individuals and communities exposed to severe forms of adversity, stress, and/or trauma (Cicchetti and Garmezy 1993; Luthar et al. 2000; Masten et al. 1990; Rutter 2012).

My research program for the past 15 years has been dedicated to advancing representation of SGD people in transdisciplinary approaches to studying stress and resilience. My goal for this article is to selectively review my groups' and collaborators' research on stress physiology, mental health, and resilience among 2S/LGBTQIA+ people. While our initial focus was on marginalization processes proposed by sexual and gender minority stress models that I will introduce next, we also wish to emphasize our increasing focus on also understanding hardiness and resilience processes of 2S/LGBTQIA+ communities around the world. Rather than attempting an exhaustive review of the rapidly expanding stress literature on SGD people, this paper adopts a depth‐over‐breadth approach by drawing on a coordinated research program from my laboratory and that of my colleagues. This approach is intentional. The aim is not to catalogue findings, but to use a set of theoretically aligned studies as a coherent “case series” to demonstrate how stress biology can be reconceptualized beyond binary frameworks. Many of these gender/sex binaries (SturtzSreetharan et al. 2025) pertain specifically to sex (male/female), gender identity (cisgender/transgender), and sexual orientation (heterosexual/non‐heterosexual). I would argue that these binaries also “conceptually contaminate” how we construct exposures (stressed/not stressed), outcomes (mental well‐being, physical health, cognitive reserve), and mediators/moderators (risk/resilience constructs). Throughout this work, I endeavor to move toward more multidimensional approaches with continuums rather than solely categories.

Taken together, this selective review will illustrate how stress biology can be operationalized in ways that better reflect variability, adaptability, and heterogeneity in lived experience of SGD populations. By foregrounding these contributions, my aim is to offer a focused, conceptually integrated presentation for moving beyond binary approaches in stress science, while situating this program of research within broader theoretical developments. Since 2018, I am privileged to mentor students and direct staff at our laboratory, the Centre d'Études sur le Sexe*Genre, *l'Allostasis*, *et la Résilience* (CESAR). At the core of our activities is the following question: “How does the social environment modulate stress physiology and what risk/resilience pathways buffer links between exposures and outcomes?” Note that our focus is not on integrating biobehavioral measures to study sexual diversity (for an excellent perspective, see Diamond (2026) in this Special Issue) or gender diversity, but rather using stress biology to examine how socio‐cultural contexts become biologically embodied.

Before we continue, I wish to make an important note on terminology that is constantly evolving among activists and academics alike. Following recent scholarship arguing for a shift away from identity‐based and context‐based categorization toward understanding sexual and gender diversity as population‐level phenomena rather than fixed “types of people” (Hammack 2025), my CESAR team and I endeavor to adapt and revise our terminology respectfully. Over the years, for example, our team has moved away from the term “sexual and gender minorities” toward “sexually and gender diverse” or SGD as a non‐identity descriptor that more accurately captures the full range of individuals affected by the stressors discussed in the narrative review. This is meant to also include those who do not identify as *transgender and nonbinary* (TNB); however, we also wish to acknowledge that TNB is sometimes the preferred term by the communities we study and serve.

My research program for the past 15 years has been dedicated to advancing representation of SGD people in transdisciplinary research on stress and resilience. Much has changed scientifically, socially, and politically in both Canada (where our CESAR laboratory is located) and the United States (where key collaborators are based and where I trained as a post‐doctoral fellow at Columbia University) during that period. In this narrative, I draw together the science emerging from our collaborative research alongside these broader sociopolitical shifts to contextualize how and why our thinking and research practices changed over time, particularly regarding binary frameworks, identity categories, and approaches to stress research. This narrative approach allows the paper to move beyond a selective review of findings and instead illustrate how scientific questions, methodological practices, and interpretations of stress and resilience evolved in dynamic interaction with changing sociopolitical realities and the lived experiences of North American and international SGD communities. Throughout, I will continually reflect on what has changed in our thinking, methods, assumptions, and interpretations over time.

## 2 Sexual and Gender Minority Stress and Resilience

The 2S/LGBTQIA+ community is highly susceptible to stress‐related disease because of stigma exposure (IOM 2011). Much of the 2S/LGBTQIA+ health literature has been informed by *sexual and gender minority stress* models (BBockting et al. 2013; Brooks 1981; Meyer 1995, 2003) that focus on two key stress processes. First, *proximal minority stress processes* refer to individual‐level experiences like internalized transphobia and concealment of one's minority identity (Herek 2009). Second, *distal minority stress processes* refer to stressors like discrimination and violence experienced at the interpersonal level. This also includes *structural stigma*, the societal‐level conditions, cultural norms, and institutional policies that constrain the opportunities, resources, and well‐being of the stigmatized (Hatzenbuehler et al. 2013).

In minority stress models, *resilience* is seen as the coping strategies and buffering effects derived from protective processes that help dampen the negative effects of stigma on health. For example, protective factors like social support strengthen health, well‐being, and quality of life of older 2S/LGBTQIA+ people (Erosheva et al. 2016; Fredriksen‐Goldsen, Kim, et al. 2017; Fredriksen‐Goldsen et al. 2015; Kim and Fredriksen‐Goldsen 2016). But these protective factors are experienced differently among 2S/LGBTQIA+ sub‐groups. For instance, bisexual people garner less social support and community resources than lesbians and gay men (Fredriksen‐Goldsen, Shiu, et al. 2017). Critically, resilience should not be treated as an ancillary construct but as a constitutive element of the minority stress process itself (Meyer 2015). Resilience co‐evolves with stress exposures as individuals and communities activate and appropriate coping responses, often surviving and even thriving despite adversity. This framing situates resilience as a partner in the stress‐to‐illness cascade, rather than a downstream state unique to a select few.

The integration of biological measures to the study of sexual and gender minority stress processes provides invaluable insights into both stress processes contributing to disease trajectories but also salubrious pathways that promote resilience. To better characterize stress‐related mechanisms whereby stigma “gets under the skin and skull,” it is my conviction that 2S/LGBTQIA+ health research must integrate objective biological measures in parallel to subjective psychosocial measures and approaches. Although it must be acknowledged that the social can often become the biological and so the distinctions among what is objective and subjective appear to be more and more blurred. Extending this integrative approach, a biocultural lens reminds us that inequality is not simply “experienced,” but biologically instantiated through processes of embodiment that unfold across time and context (Dressler et al. 2005; Gravlee 2009; Kuzawa and Sweet 2009). In this sense, integrating stress biomarkers with psychosocial measures collectively allows us to more fully capture how stigma and resilience manifest into the health and wellness trajectories of 2S/LGBTQIA+ populations.

## 3 Stressors, Stress, and Stress Responses

During my undergraduate studies with Jennifer McGrath at Concordia University and my graduate studies with Sonia J. Lupien and Jens C. Pruessner at McGill University, I was introduced to and inspired by perspectives advocating for the integration of multiple traditions in stress research to promote transdisciplinary research that combined sociological, psychological, and biological traditions (Juster et al. 2011). Stress is typically conceptualized as a real or perceived threat to physiological or psychological integrity that elicits coordinated behavioral and biological responses (McEwen and Seeman 1999). Contemporary models stemming from psychoneuroendocrinology emphasize that stress is multidimensional, involving interactions among environmental exposures, subjective appraisals, and downstream biological responses (Levine 2005; Levine and Ursin 1991).

Biobehavioral stress responses are primarily mediated by activation of the *sympathetic‐adrenal‐medullary* (SAM) and *hypothalamic–pituitary–adrenal* (HPA) axes, resulting respectively in the release of catecholamines like adrenalin as well as glucocorticoids like cortisol (McEwen and Wingfield 2003; Sapolsky 2004; Sapolsky et al. 2000). Cortisol secretion follows a robust diurnal rhythm, characterized by a peak shortly after waking known as the *cortisol awakening response* (CAR) and a gradual decline across the day (Pruessner et al. 1997; Stalder et al. 2015, 2022). In addition to basal rhythms, cortisol reactivity can be assessed using standardized stress reactivity paradigms.

From a developmental perspective, Shonkoff et al. (2009) differentiated stress physiology using a useful taxonomy: (1) *positive stress* refers to moderate stress responses that are normal, short‐lived, and appropriately deactivated; (2) *tolerable stress* refers to potentially damaging stress responses that get buffered and can even promote protection against future stressors; and (3) *toxic stress* refers to damaging stress responses occurring under conditions of severe adversity, stress, and/or trauma where there is a lack of internal and/or external resources to cope (Shonkoff et al. 2009). Taken together, these multidimensional processes are particularly relevant for SGD populations, for whom stigma‐related exposures may operate as chronic and context‐dependent stressors. Building on this foundation, we next consider how repeated stress exposure becomes biologically embodied through dynamic processes of allostasis and allostatic load, with particular attention to application to the study of SGD populations.

## 4 Allostasis, Allostatic States, and Allostatic Load

Stress physiology is a potential mechanism linking 2S/LGBTQIA+ stigma to physical and mental health disparities (Lick et al. 2013). As previously introduced, stressful conditions are related to measurable physiological functions that are often then correlated to other biopsychosocial phenomena. The neurobiologist Paul Sterling—brother of Anne Fausto‐Sterling who contributes brilliantly to this Special Issue (Fausto‐Sterling 2025)—and the epidemiologist Jospeh Eyer coined the term allostasis to describe dynamic, multi‐faceted biological processes that maintain physiological stability by recalibrating homeostatic parameters and matching them appropriately to meet environmental demands (Sterling and Eyer 1988). Collectively, SAM‐ and HPA‐axis stress responses are examples of *allostasis*, defined as adaptive biological processes that preserve “stability through change” (Sterling and Eyer 1988). In contrast to homeostasis, allostatic processes alter physiological functioning via compensatory and anticipatory mechanisms (Juster, Russell, et al. 2016; Juster, Seeman, et al. 2016).

In this manner, allostasis fundamentally differs from homeostasis because of its emphasis on dynamic rather than static biological set‐points, considerations of the brain's role in feedback regulation (Ganzel et al. 2010), as well as its view of health as a whole‐body adaptation to contexts (Schulkin 2003). Health in allostatic theory is defined as optimal predictive fluctuation, whereby shifts in the probability of demand should shift the response. When the prediction reverses, so should the response (Bizik et al. 2013). It is when allostasis is chronically dysregulated and energy depleted or oversupplied that pathophysiological processes emerge (Bobba‐Alves et al. 2022). Over time and cumulative use, stress responses and other allostatic mechanisms can become dysregulated via (A) *repeated activations*, (B) *lack of adaptation*, (C) *prolonged responses*, or (D) *inadequate responses* that are referred to as *allostatic states* (Koob and Le Moal 2001). Social adversity (e.g., stigma) can strain allostatic processes to induce a multi‐systemic “domino effect” that strains inter‐connected physiological systems (Juster, Seeman, et al. 2016).

*Allostatic load* (AL) represents the multi‐systemic “wear and tear” of chronic stress in synergy with health‐related behaviors (McEwen and Stellar 1993) like poor diet, sleep problems, and physical inactivity (Suvarna et al. 2020). AL reflects the dynamic functioning of multiple biological systems that are intricately interconnected to (epi)genetic, neurological, developmental, behavioral, cognitive, and socio‐cultural factors. For more than 30 years, the allostatic load model has strongly shaped stress science. As of April 1st, 2026, “allostatic load” returns 2875 hits on PubMed and 70 700 hits on Google Scholar. AL is measurable by using a battery of biomarkers representing *neuroendocrine* (e.g., cortisol), *immune*, *metabolic*, and *cardiovascular* systems (McEwen and Stellar 1993; Seeman et al. 1997). AL indices predict numerous health outcomes better than traditional biomedical approaches because algorithms ascribe sub‐clinical thresholds for *several* biomarkers (Juster et al. 2010). For example, AL has been consistently associated with accelerated aging (Crimmins et al. 2003), lower socio‐economic status (Crimmins et al. 2009), minority race/ethnic status (Merkin et al. 2009) and numerous health outcomes and mortality (Sabbah et al. 2008).

**FIGURE 1:**
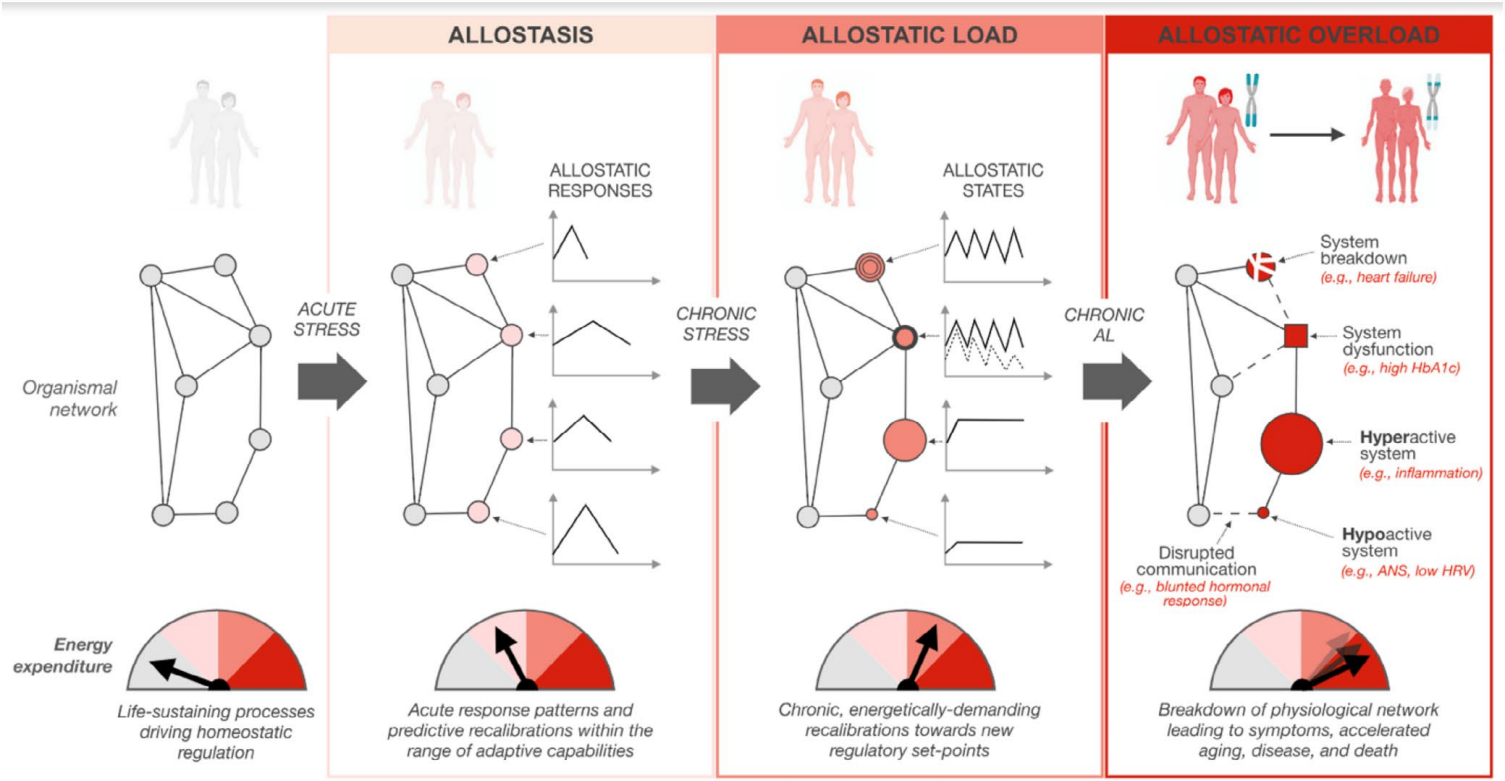
The energetic basis of the allostatic load stress–disease cascade. A physiological network of interconnected systems (nodes) and their communication (edges) operates under biopsychosocial and environmental influences. Changes in node appearance reflect dysfunction and altered activation (hypo/hyper) under allostatic load, while dashed lines indicate impaired communication. These disruptions emerge as circulating biomarkers and, over time, clinical symptoms. Allostatic states are adapted from McEwen's time‐course model. *Source:* Bobba‐Alves et al. (2022).

From an evolutionary perspective, *allostatic overload* represents the AL breaking point (Figure 1). *Type 1 allostatic overload* occurs when energy demands exceed energy supply (negative energy balance), for example, when starving or migrating (McEwen and Wingfield 2003). Such situations activate an emergency life history stage to help cope with the needs of survival (Wingfield et al. 1998). This can be thought of as a protracted allostatic state under acute conditions. By contrast, *Type 2 allostatic overload* occurs when energy supplies exceed energy demand (positive energy balance), for example, when overeating or hibernating. Overtime, strained allostatic responses shift functioning from AL to allostatic overload in synergy with changes in health‐related behaviors like sleep, exercise, diet, drugs, and alcohol (Suvarna et al. 2020). Taken together, we can distinguish AL as it relates to a spectrum ranging from a normal adaptation to predictable environmental demands that can, in unfavorable conditions, lead to the two varieties of allostatic overload (McEwen and Wingfield 2003).

## 5 Indexing Allostatic Load

Beyond theory, measuring AL is possible by indexing multiple biological dysregulations. It is important to note AL has been criticized on theoretical, empirical, and clinical grounds (Beckie 2012; Finlay et al. 2026; Juster and Misiak 2023) that I will elaborate upon further on. Notwithstanding, the AL model continues to grow in popularity and expands into numerous new health areas like oncology and artificial intelligence. Beyond informing stress theory, AL measurement has been made possible using AL indices that have been well‐validated and applied across lifespan development in now well over 300 international studies. Most commonly, AL is calculated by using the traditional *sample‐based AL formulation* that sums the number of dysregulated biomarkers using high‐risk cut‐offs: the 75th percentile for biomarkers where high levels are damaging or the 25th percentile where low levels are damaging. In a few exceptions, a two‐tailed approach is also common (12.5th percentile or 87.5th percentile) for biomarkers like cortisol where both hypo‐ and hyper‐functioning can be problematic. AL therefore represents the number of dysregulated biomarkers.

Alternatively, a *sex‐specific AL formulation* uses the sample‐based approach but with cut‐offs that are specific to each birth‐assigned sex separately (Juster, Preussner, et al. 2016). In the general population, birth‐assigned males tend to have higher AL than birth‐assigned females (Kerr et al. 2020). To best capture this, I have advocated that a sex‐specific formulation can help reduce this variance if the study design requires it, such as when investigating sex‐specific and/or gender‐based variability. A major focus of future research endeavors with Zachary DuBois is to advance *gender‐specific AL indices* that represent biological functioning according to gender identity. In this manner, we can envision calculating AL indices, for example, for transgender, nonbinary, gender, and cisgender people separately. This is critically important as clinical norms are often based exclusively, if at all, on male and female ranges without consideration of gender diversity. Collectively, we believe that these various AL algorithms, and many more in the literature (Longpre‐Poirier et al. 2022), can help account for sex differences and gender diversity in biomarkers and AL and then allow us to assess the role of stress‐ and resilience‐related processes among diverse populations.

The AL model's problems compound across levels of criticism that I wish to acknowledge. Theoretically, the cascade it presumes (e.g., primary mediators dominoing into secondary and tertiary dysregulation) remains only partially confirmed, with a complex interplay among physiological systems undercutting the linear logic the model was built on. Thanks to the seminal work of my colleague Martin Picard at Columbia, a refined AL model that is reframed energetically (see Figure 1) looks less like dysregulation and more like a tradeoff where allostasis competes with growth, maintenance, and repair for finite resources (Bobba‐Alves et al. 2022; Picard et al. 2014, 2017). Empirically, the model is still hostage to its own operationalization: a persistent lack of consensus limits comparison across studies, so AL indices built from different biomarkers and cutoffs may not be measuring the same thing. Clinically, AL remains more descriptive than actionable: applied work documents exposure and awareness without testing whether intervention changes anything, leaving “theory to therapy” a slogan more than a pipeline (Juster and Misiak 2023).

## 6 Generalized Unsafety Theory of Stress

Despite criticisms, the AL model and associated algorithms are constantly evolving as part of the broader stress literature. More recently, theoretical developments in health psychology have challenged core assumptions underlying traditional stress models. Notably, the *generalized unsafety theory of stress* (GUTS) proposes that the human stress response system may not default to a state of homeostatic or energetic “calm”, but rather to a baseline condition of generalized unsafety in the absence of sufficient safety cues (Brosschot et al. 2018a, 2018b). From this perspective, sustained physiological activation does not necessarily require discrete or repeated stressors, but may instead reflect the chronic absence of perceived safety, such as in the case of discrimination (Acevedo et al. 2020).

This contrasts with the AL framework, which emphasizes the cumulative “wear and tear” resulting from repeated and/or prolonged stressor exposure across biological systems (McEwen and Seeman 1999; McEwen and Wingfield 2003). While GUTS provides an important reconceptualization of baseline stress physiology, particularly in contexts of chronic uncertainty or low environmental safety, AL remains a widely used heuristic platform for operationalizing multisystem biological risk in population health research. In my view, these perspectives may be viewed as complementary, with GUTS refining assumptions about tonic physiological activation and AL capturing downstream patterns of biological embodiment.

Taken together, I consider these developments not as a repudiation of the AL model. Rather, these innovations are evidence of an evolving field that is actively refining how we conceptualize and operationalize stress biology. Admittedly, GUTS offers an important conceptual advance by challenging the assumption of a quiescent physiological baseline, instead foregrounding the possibility that chronic activation may reflect persistent states of unsafety rather than discrete stressor exposure. At the same time, AL retains enduring heuristic and integrative value as a biobehavioral framework that captures the cumulative physiological consequences of lived experience across neuroendocrine, immune, metabolic, and cardiovascular systems.

Importantly, longstanding critiques regarding biomarker selection, weighting, and construct validity underscore that AL is not a static endpoint (McCrory et al. 2023). Instead, AL algorithms are an evolving toolkit that must be applied thoughtfully, particularly in diverse populations (Guidi et al. 2021; Juster and Misiak 2023; Whelan et al. 2021). Integrating new stress models such as GUTS with increasingly nuanced AL formulations that include sex‐ and gender‐informed approaches positions the field to more precisely tease apart how social conditions become biologically embodied. This is especially critical when turning to the study of sexual and gender minority populations who face chronic exposure to stigma, discrimination, and structural inequities that may fundamentally shape baseline physiology and the probabilistic pathways linked to health trajectories over time.

## 7 Stress Physiology and Sexual Diversity

My research program has applied cortisol and AL measurement to the comprehensive study of 2S/LGBTQIA+ health with studies first among sexual minorities (Juster, Ouellet, et al. 2016; Juster et al. 2013). In most of my research projects to date, participants are exposed to the *Trier Social Stress Test* (Kirschbaum et al. 1993). Participants are also asked to sample saliva over the course of the day to measure diurnal cortisol (e.g., awakening, +30 min after, bedtime). To account for sex‐based variations in circadian profiles, our group systematically collects salivary gonadal hormones (*testosterone*, *dehydroepiandrosterone*, *estradiol*, and *progesterone*) in all participants at baseline for use as covariates (Juster, Preussner, et al. 2016; Juster, Raymond, et al. 2016) and to explore inter‐correlations with other study variables. This approach adjusts for individual differences that are generally ignored in the stress literature. In addition, this allows for a continuous measure of hormones comprising the construct of biological sex that could otherwise confound cortisol.

**FIGURE 2:**
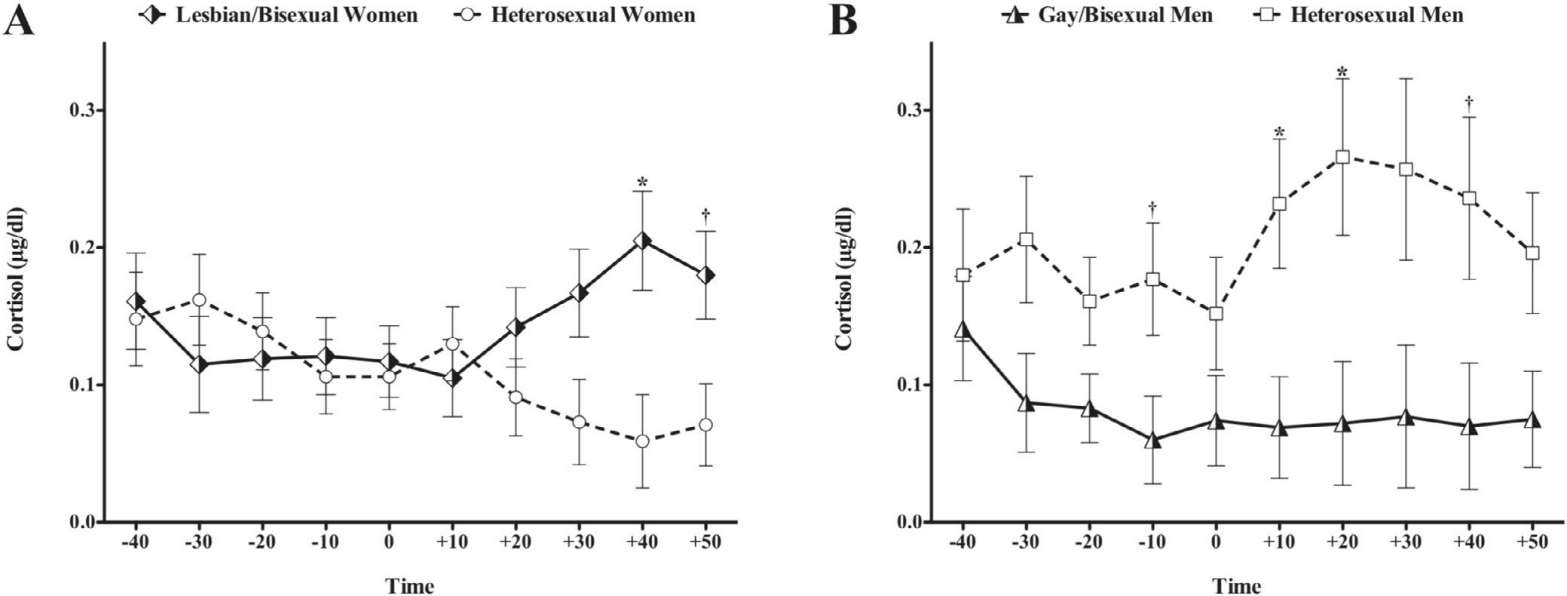
Cortisol concentrations in response to the Trier Social Stress Test among (A) women (*n* = 40) and (B) men (*n* = 46) as a function of sexual orientation. *Source:* Juster et al. (2015).

During my graduate studies with Sonia J. Lupien and Jens C. Pruessner, we provided data emphasizing the importance of sexual orientation when studying reactive cortisol, diurnal cortisol, and AL. In a first study of Montrealers between 18 and 45 years old (mean age 25), we assessed cortisol stress reactivity, diurnal cortisol variation, AL, and mental health as a function of sexual orientation. In accordance with sexual minority stress theory, we hypothesized that lesbian, gay, and bisexual participants would have stress‐related profiles worse off than heterosexual participants. That is, we expected them to have dysregulated cortisol profiles, high AL, and poor mental health consistent with our understanding of distal and proximal stress processes. But much to our surprise, we found the opposite patterns. As seen in Figure 2, our findings showed that compared to heterosexuals, gay and bisexual men had less cortisol reactivity but higher heart rate (Juster et al. 2019) in response to the psychosocial stressor, but lesbian and bisexual women had higher cortisol reactivity (Juster et al. 2015). Sexual minority women also showed higher androgens and progesterone that correlated with psychosocial stress (Juster, Almeida, et al. 2016). Interestingly, among sexual minority women, higher testosterone and progesterone predicted greater cortisol stress reactivity. While replication is required, this convergence of associations demonstrated the importance of measuring sexual orientation in stress reactivity studies. At the time of this work, we had not included TNB people who were far less represented in health research at this time (2010–2015). Since then, my CESAR team has actively focused on gender diversity and inclusion of TNB people with new data that we will be presenting and publishing soon.

**FIGURE 3:**
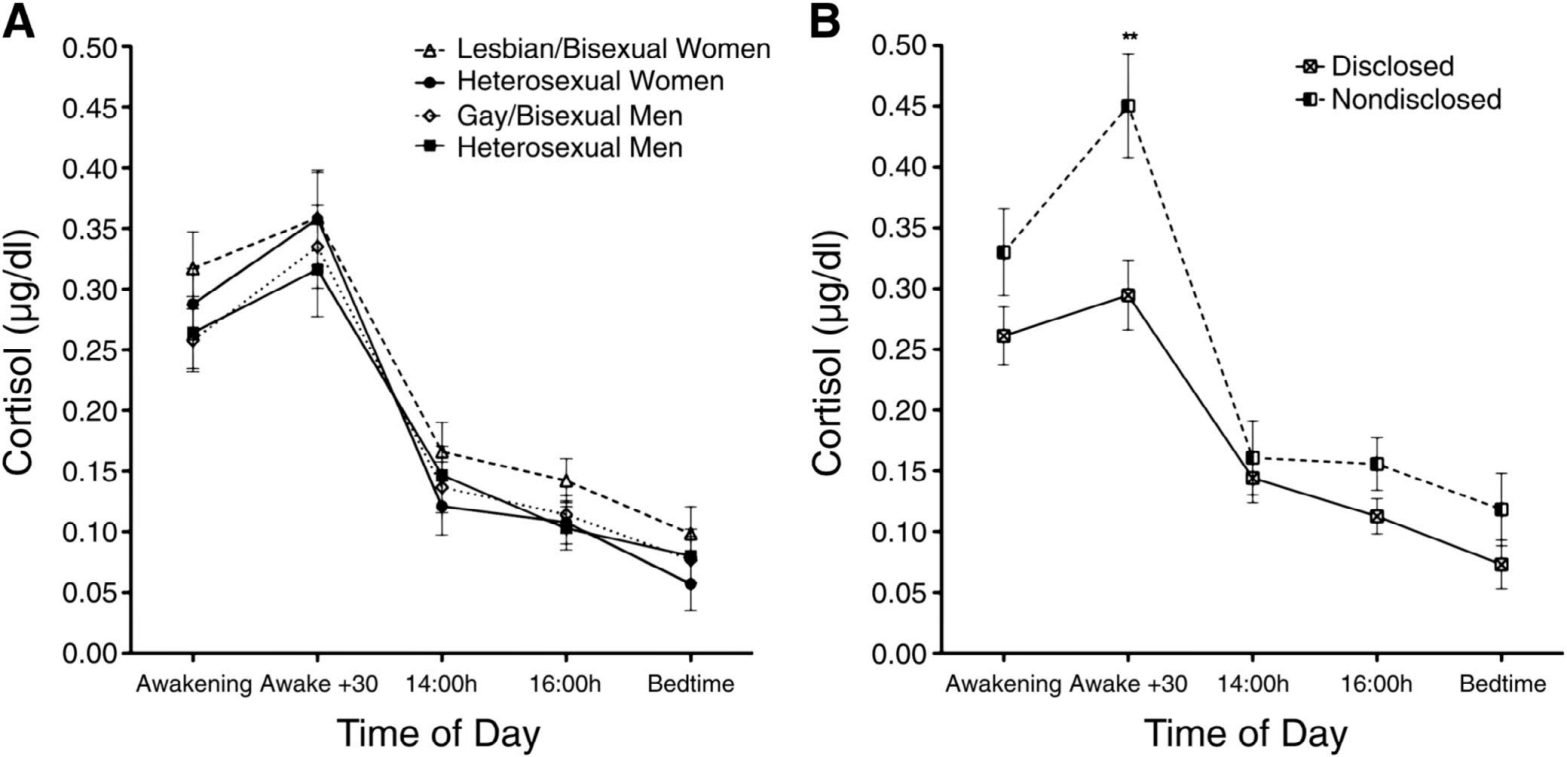
Salivary diurnal cortisol concentrations for two averaged days as a function of sexual orientation and sex while controlling for chronic stress and awakening time (A) and for disclosed and nondisclosed sexual minorities (B). *Source:* Juster et al. (2013).

**FIGURE 4:**
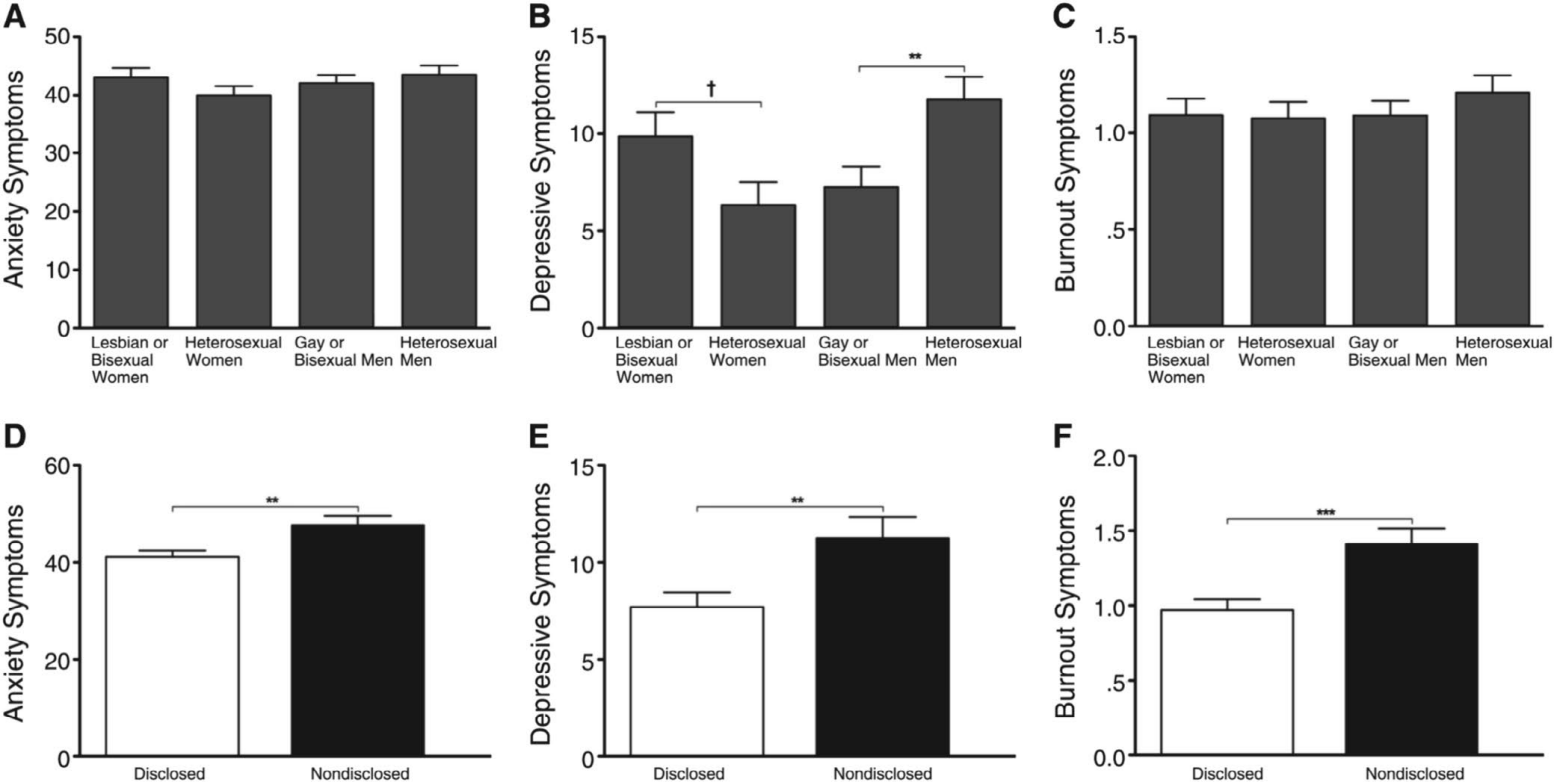
Mental health as a function of sexual orientation and sex and disclosure status. First, anxiety symptoms (A), depressive symptoms (B), and burnout symptoms (C) among male and female sexual minorities and heterosexuals are depicted. Second, anxiety symptoms (D), depressive symptoms (E), and burnout symptoms (F) among disclosed and nondisclosed sexual minorities are depicted. *Source:* Juster et al. (2013).

**FIGURE 5:**
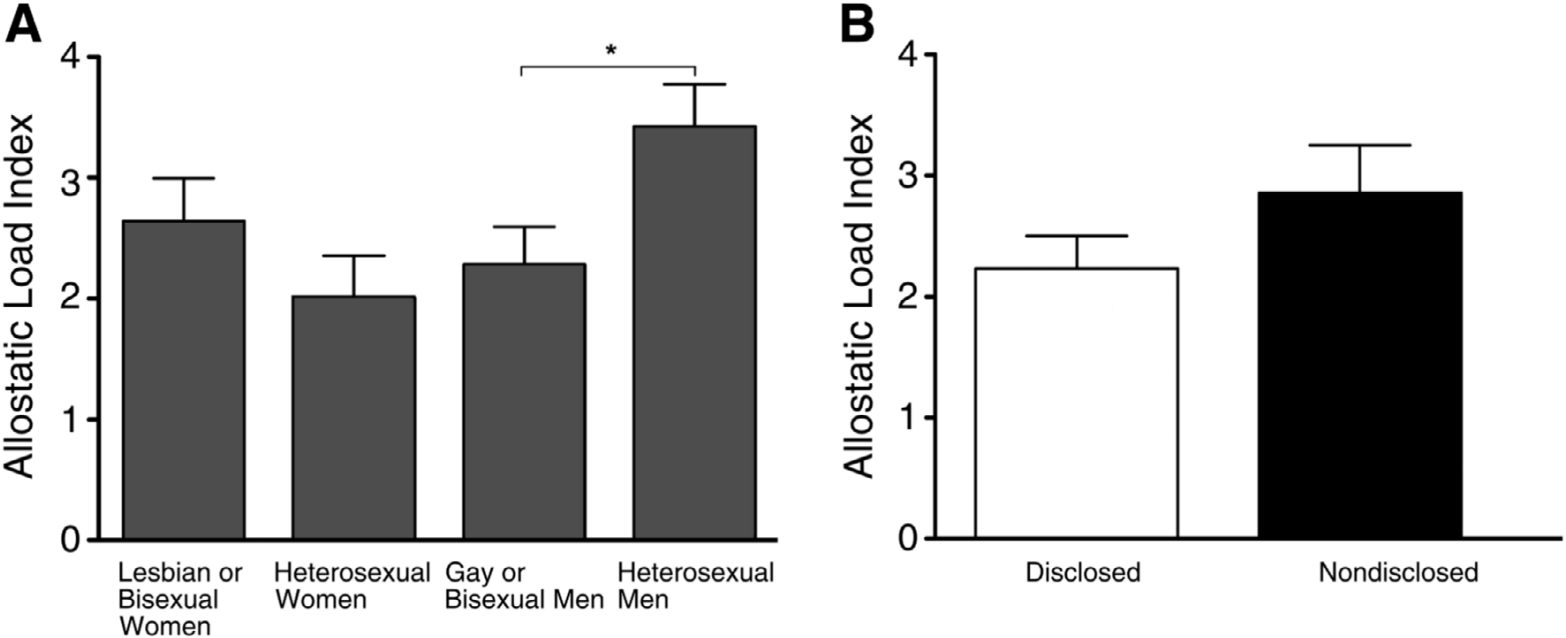
Allostatic load indices based on 21 biomarkers as a function of sexual orientation and sex (A) and for disclosed and nondisclosed sexual minorities (B). Montreal sample (*N* = 87). *Source:* Juster et al. (2013).

Beyond differences according to sexual orientation, navigation of proximal stress processes had a strong influence on HPA‐axis diurnal functioning and mental health (Figure 3). Specifically, LGB people who had “come out” to their families and friends showed lower levels of morning cortisol than those who had not completely disclosed their sexual identities (Juster et al. 2013). “Coming out” was also associated with better mental health across the board: less symptoms of anxiety, depression, and burnout (Figure 4). Lastly, using an AL index consisting of 20 biomarkers, we were also surprised that LGB people did not have elevated AL consistent with sexual minority stress theory (Figure 5). In fact, gay men had *lower* AL levels (Juster et al. 2013) than heterosexual men. This was driven by less triglycerides, lower body mass indices, and a trend toward less inflammation among gay and bisexual men as compared to heterosexual men. By contrast, women showed no differences in AL by sexual orientation (Juster et al. 2013).

In summary, these findings were somewhat inconsistent with central tenets of sexual minority stress theory. In a review of 26 studies assessing the link between sexual minority stress and biomarkers, 42% of studies showed relationships while 58% of studies did not (Flentje et al. 2020). Likewise, in this first Montreal study, we did not find evidence that AL was higher in sexual minorities. Given that the “coming out” process was associated with lower HPA‐axis output and better mental health, we suspect that this hardy sample of LGB Montrealers might be showing some resilient profiles. The reader will recall that LGB‐specific stress processes are also associated with unique coping behaviors that bolster resilience. Indeed, specific patterns of risk in combination with protective factors may be uniquely utilized among LGB sub‐groups given their adaptations to stigma across life. In particular, health‐related behaviors are appropriated differently by sub‐groups, with gay men and lesbian women showing a mix of both positive and negative profiles that will be elaborated upon further on.

To assess potential resilience, we further explored coping strategies in relation to AL and mental health in secondary data analysis of this Montreal sample. Central to integrating one's self‐identity as a sexual minority is developing strategies to help cope with the stressful realities of a marginalized identity. When looking at coping strategies, lesbian, gay, and bisexual young adults who engaged in less avoidance regarding their sexual orientation showed lower AL and those who sought social support reported less perceived distress (Juster, Ouellet, et al. 2016). Collectively, this suggests that individual (e.g., coping strategies) and interpersonal (e.g., disclosure, social support) factors may promote resilience to AL and psychiatric symptoms among lesbian, gay, and bisexual Canadians in our Montreal sample.

**FIGURE 6:**
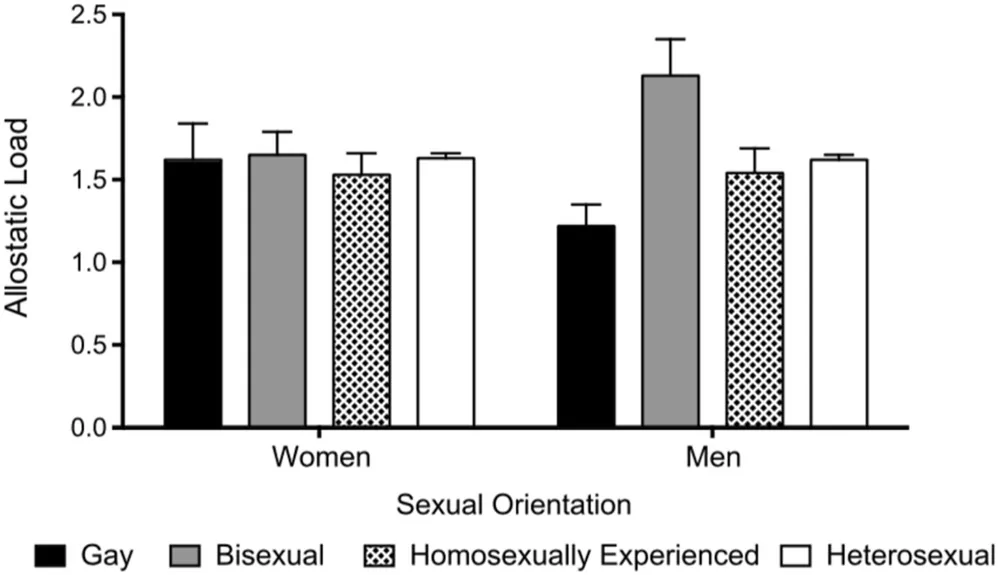
Allostatic load as a function of sexual orientation stratified by sex in the *National Health and Nutrition Examination Survey* from 2001 to 2010 (*N* = 13 911). *Source:* Mays et al. (2018).

To confirm whether these AL findings could generalize to the United States in a larger sample ages 20 to 59 (mean age about 40 years old), a UCLA collaborative project led to an analysis of the *National Health and Nutrition Examination Survey* or NHANES (Mays et al. 2018). Consistent with original findings from Canada, gay men showed lower AL than heterosexual men. By contrast, bisexual men showed the highest AL among men and women. AL again did not differ for women by sexual orientation, perhaps because the clinical norms used to calculate AL ignored sex differences in biomarker values (Juster, Preussner, et al. 2016). We replicated our finding of lower AL in gay men using a much larger American sample (Figure 6). Again, however, no differences in AL were detected as a function of sexual orientation. This was somewhat perplexing and again inconsistent with sexual minority theory.

**FIGURE 7:**
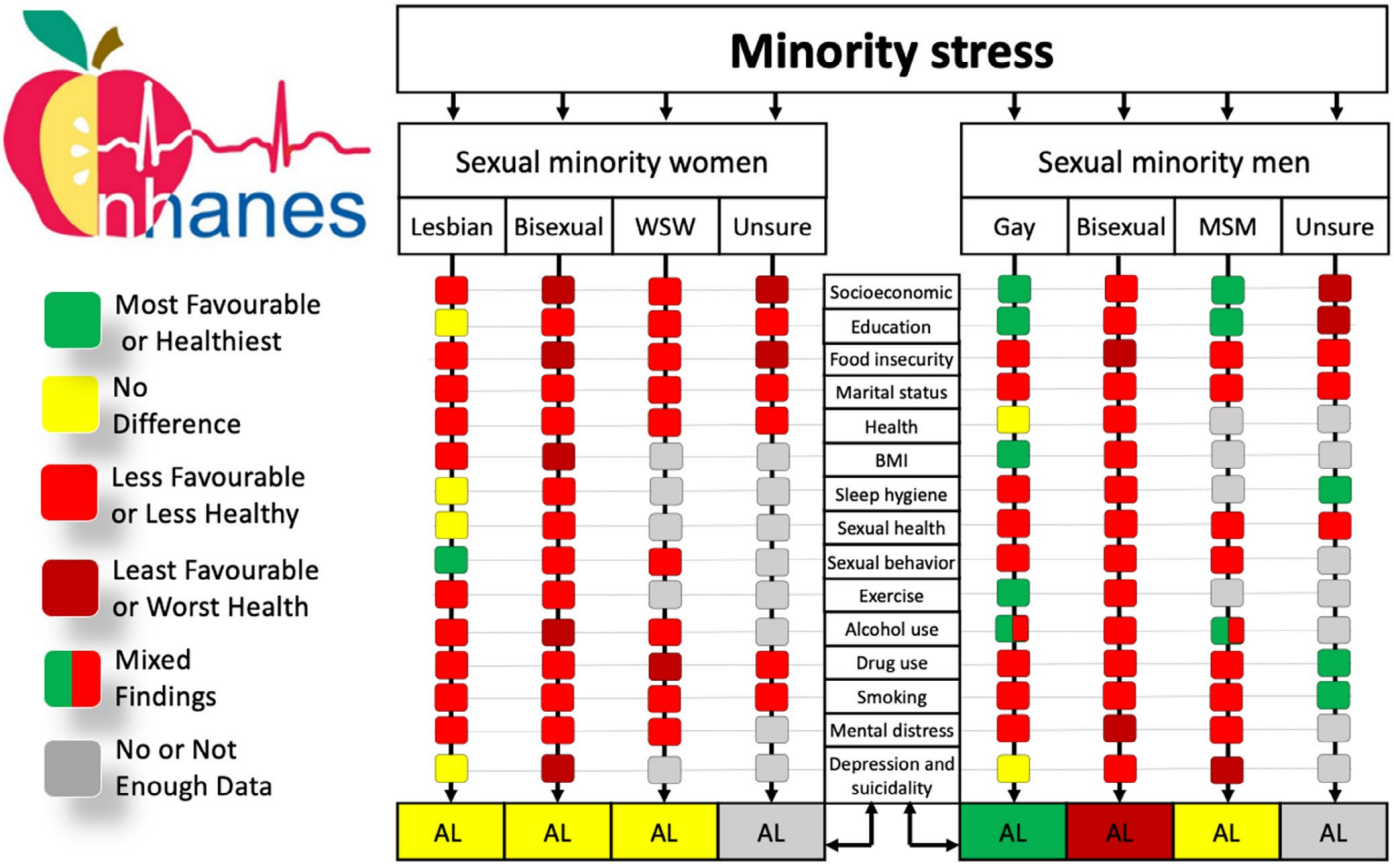
Summary of National Health and Nutrition Examination Survey (NHANES) studies (*N* = 43) that assess sexual orientation/behaviors and health‐related variables. Associations are color coded according to the left legend. Abbreviations: AL, allostatic load; BMI, body mass index; MSM, men who have sex with men; WSW, women who have sex with women. *Source:* Desjardins et al. (2022).

What could explain these lower AL levels observed in young gay men, no differences among women, and higher AL in bisexual men? In a review of 42 studies on sexual orientation that used the NHANES (Desjardins et al. 2022), gay men showed a mix of protective profiles (e.g., lower body mass index, higher education) in addition to some risk profiles (e.g., more sexually transmitted). Similarly, lesbian women show some salubrious pathways in combination with risk profiles (Figure 7). Strikingly, both bisexual women and bisexual men show negative profiles (Desjardins et al. 2022). This pattern of high‐risk among bisexual people could explain why bisexual men have the highest AL in this NHANES analysis of nearly 14 000 Americans. These patterns of differential sociodemographic, health‐related behaviors, and other psychosocial characteristics are very different from one LGB sub‐group to the other. This highlights the need for more granular analyses in population‐based studies since each sub‐group is appropriating unique mixes of risk and protective factors that are not captured when clumped together in to a single LGB monolith.

Collectively, these studies provided the first evidence of sexual orientation differences in Canadians and Americans, with young gay men showing the lowest AL. Canada and the United States are similar yet distinct societies with different systems of structural stigma. In contrast to the American context, Québec became the first North American jurisdiction to prohibit discrimination against sexual minorities by amending the Charter of Human Rights and Freedoms in 1977, and Canada legalized same‐sex union across the nation with the Civil Marriage Act in 2005. Québec and Canada have therefore been champions of protective policies for 2S/LGBTQIA+ people. At this time in 2026, North America is undergoing geopolitical changes that necessitate further research evidence. This makes the comprehensive measurement of stress biomarkers like cortisol measurement and AL indices a crucial endeavor, providing us with objective biometrics. From a public health perspective, these biometrics can then be correlated to macro‐level effects that can inform policy makers of the pernicious effects of institutionalized stigma and how to improve the health of marginalized groups.

Understanding stress biomarkers in health inequalities requires a macro‐level perspective. Social inequalities impact health but can be mitigated through policy changes (Hatzenbuehler 2010). Research shows that LGB individuals in states without protective policies face higher rates of psychopathology than those in states with protections (Hatzenbuehler et al. 2009). Before nationwide marriage equality, LGB individuals in states with same‐sex marriage bans experienced higher rates of anxiety, depression, and alcohol abuse, while those in states recognizing same‐sex marriage did not (Hatzenbuehler et al. 2010). Policy changes improve sexual minority health. After Massachusetts legalized same‐sex marriage, gay men had fewer medical and mental health visits and lower healthcare costs within a year (Hatzenbuehler et al. 2012). This highlights how policy shifts can reduce institutionalized stigma and enhance public health (Hatzenbuehler et al. 2012).

**FIGURE 8:**
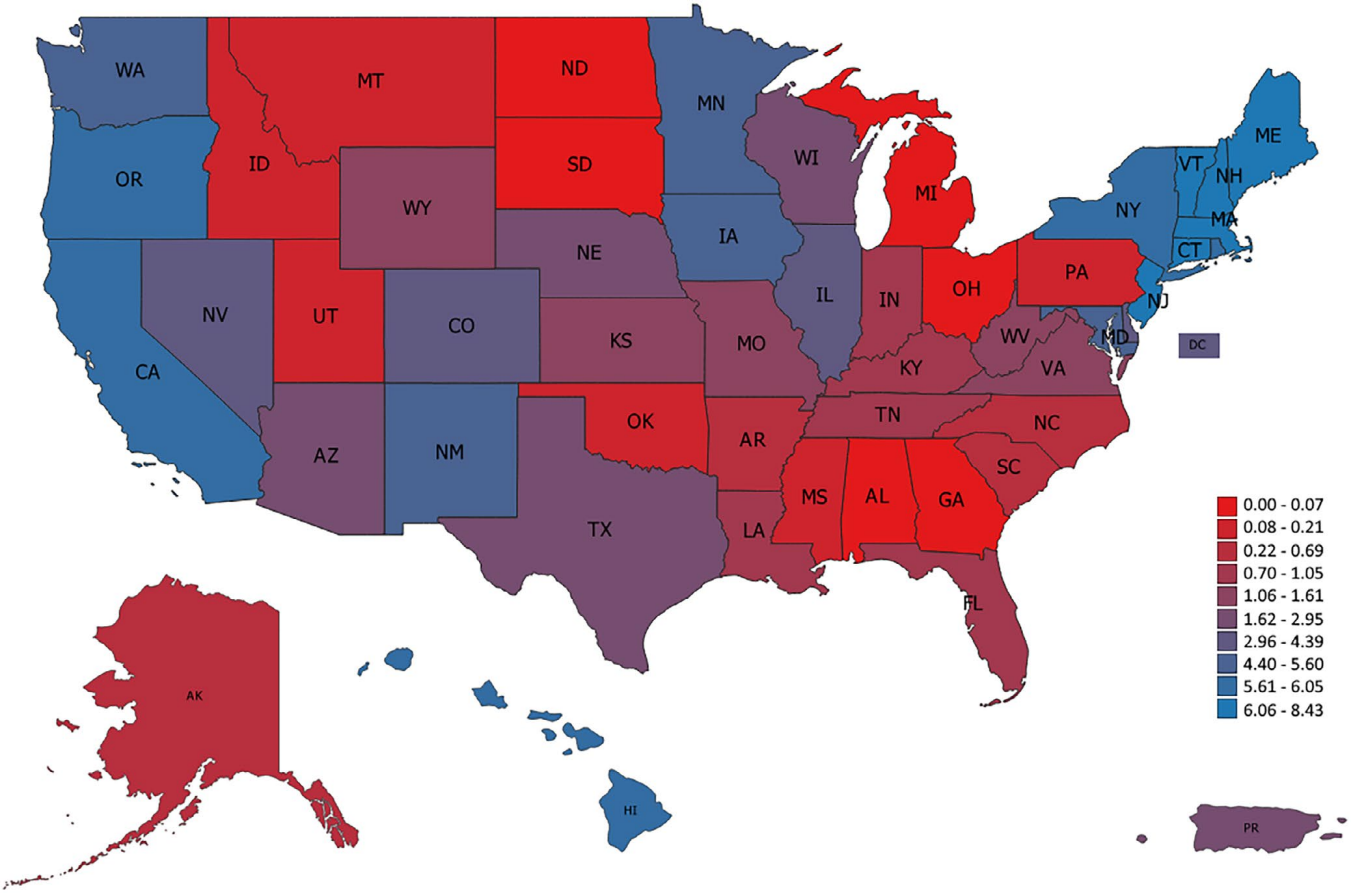
This map represents the average number of 10 state‐level policies protecting LGB Americans across the waves (2001–2014) of the National Health and Nutrition Examination Survey (NHANES). States in lighter red have higher average levels of structural stigma during this period, as indicated by fewer state‐level policy protections for LGB individuals. States in lighter blue have more state‐level policy protections for LGB individuals and hence lower average levels of structural stigma. States in purple have moderate levels of structural stigma. This map does not correspond to the statistical analyses, which treated structural stigma as time‐varying. LGB = lesbian, gay, and bisexual. Thanks to Daniel Raillant‐Clark, this shape file was created using contours provided by the US Census Bureau in conjunction with the QGIS software (https://www.qgis.org/en/site/). *Source:* Juster et al. (2024).

My post‐doctoral research working with Mark Hatzenbuehler then at Columbia University and now at Harvard aimed to assess AL levels across U.S. states with and without protective policies to examine the link between social policy and biological processes. We hypothesized that lesbian, gay, and bisexual Americans living in low‐stigma environments would show lower AL than those in less progressive regions. Using again the NHANES from 2001 to 2014, we were able to index each state according to 10 different policies related to sexual orientation as our measure of structural stigma (Juster et al. 2024). In a combined sample of about 24 000 Americans, we found that gay and bisexual men living in states with more protections had lower AL while controlling for key sociodemographics. By contrast, no significant effects were found among women by sexual orientation (Figure 8).

We suspect that the inability to identify an effect of structural stigma related to sexual orientation among women is that *structural sexism* (Homan 2019) might be more salient for cisgender women and should therefore be addressed in the future. Like structural stigma, structural sexism is related to *institutionalized gender* that refers to the ways that gender is constructed within large social systems that dictate value systems, social class, and hierarchies of privilege (Johnson et al. 2007). We suspect that there are important structural inequalities for women that supersede sexual orientation‐related stigma in its importance on effecting AL among women. This is speculative but warrants further investigation with more sophisticated sociological approaches. In addition to structural sexism or structural homophobia, structural stigma related to transphobia has also not been explored nor has gender identity in these national surveys that have just recently been added to survey cycles. This is why it is essential that SOGI items remain part of national surveys and included in federally funded research. In summary, these first studies highlight important sub‐group differences in cortisol functioning, AL, and mental health of sexually diverse North Americans.

## 8 Stress Physiology Among Transgender and Nonbinary People

Gender identity refers to a person's deeply felt sense of being a boy/man, girl/woman, both, neither, or somewhere along the gender spectrum. *Transgender and nonbinary* (TNB) people face important health disparities due to ultra‐high stigma exposure from societies that often denigrate gender nonconformity and diverse gender expressions (IOM 2011; National Academies of Sciences 2020). Alarmingly, transgender people have one of the highest rates of attempted suicide: 40%–50% compared to 2%–9% for the general population (James et al. 2016; Nock et al. 2008; Perez‐Brumer et al. 2017; Toomey et al. 2018). Distress related to stigma and the experience of discordance between one's gender identity and birth‐assigned sex is referred to as *gender dysphoria* that can be relieved through the process of gender transition by social, legal, and medical means. This can include administration of gender‐affirming hormonal therapies that often help improve mental health (Colton Meier et al. 2011; IOM 2011).

**FIGURE 9:**
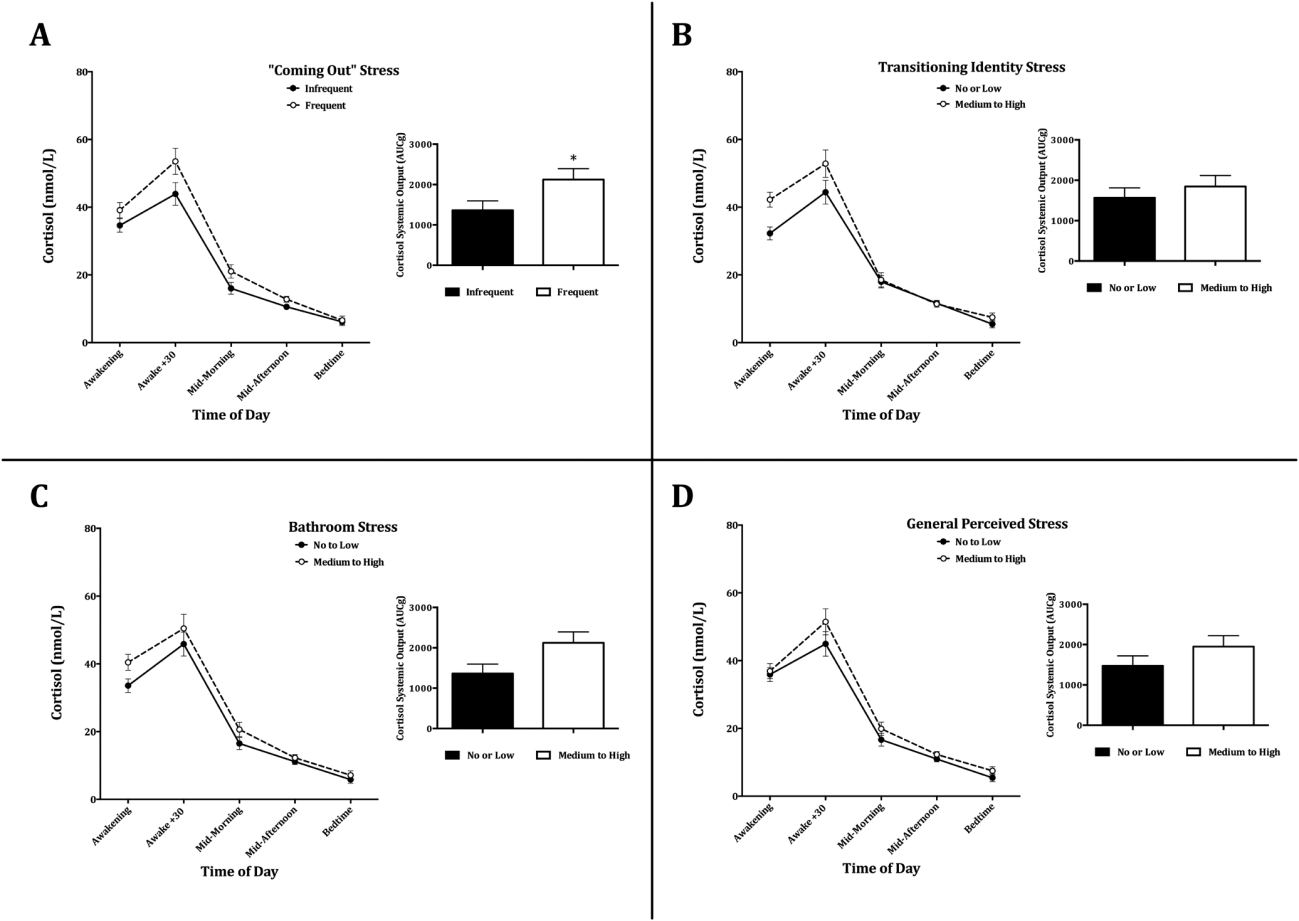
Estimated mean (±SE) diurnal cortisol as a function of transitional factors. Specifically, cortisol diurnal trajectories are shown for illustrative purposes as a function of (A) coming out stress, (B) transitioning‐identity stress, (C) gender‐specific Public Bathroom stress, and (D) perceived stress. *Source:* DuBois et al. (2017).

Pioneering research by my longtime collaborator Zachary DuBois, and the work of the Stress, Adaptation, and Resilience Lab at the University of Oregon has provided compelling research on how stigma exposure and the experience of gender affirming transition influences the stress physiology and AL of transgender people. DuBois' work has contributed significantly to biocultural approaches (DuBois et al. 2021; Dubois, Puckett, et al. 2022; DuBois, SturtzSreetharan, et al. 2022) to working with and for TNB communities in participatory research that empowers the community. In a first HPA‐axis study of transitioning transgender men (Figure 9) from New England (Massachusetts, Vermont, Rhode Island), DuBois et al. (2017) showed that those experiencing high levels of distress in relation to transition‐related stressors, “coming out” processes, and access to public restrooms showed elevated levels of diurnal cortisol. Likewise, more stress regarding “coming out” and being recognized as their affirmed gender was respectively associated with diminished nocturnal decline in blood pressure and elevated C‐reactive protein (Dubois 2012). Akin to my graduate work among sexual minorities from Montreal, these findings show that proximal stress processes like disclosure and social stressors are associated with stress‐related physiological markers among transgender men.

**FIGURE 10:**
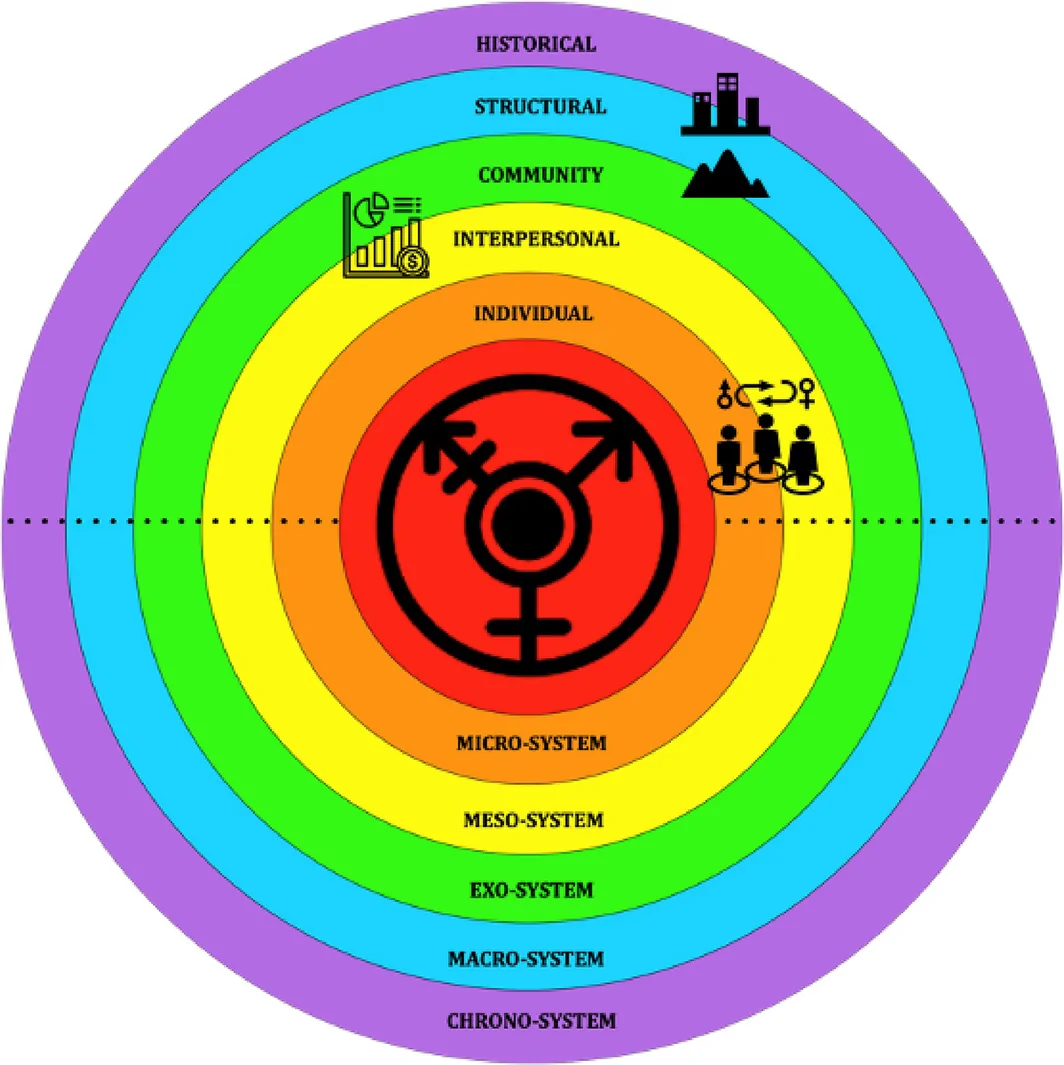
Conceptual model of lived experience combining gender minority and marginalization stress processes (top half) embedded within ecological systems (bottom half) spanning the micro‐level (immediate environment), meso‐level (interconnections among several individuals), exo‐level (the indirect influence of social structures and settings), macro‐level (current overarching cultural and sub‐cultural patterns), and chrono‐level (time period). In parallel, stress processes and stigma are experienced at individual (e.g., self‐stigma, disclosure), interpersonal (e.g., abuse, rejection, discrimination), community (e.g., marginalization), and structural (e.g., state policies, institutional practices) levels through history. These gender minority stressors depicted in the top half can be considered as compounding and in addition to those experienced everyday by many people. *Source:* DuBois and Juster (2022).

In combining several biomarkers in a mixed method analysis, DuBois and Juster published the first AL study among transgender men from the same New England sample. Using a systems socioecological model, we used interview and questionnaire data to index health‐related behaviors, transgender‐specific stressors, social support, socio‐demographic advantage, and progressive geopolitical climate. We found that transmasculine people living in less geopolitically progressive spaces and with less sociodemographic advantage showed elevated AL (DuBois and Juster 2022). Mental health (anxiety, depression, and insomnia) was more strongly associated with transgender‐specific stressors. Our approach of assessing micro‐, meso‐, and macro‐level domains is a promising way to contextualize lived experiences (Figure 10), especially with smaller samples. Given the ongoing challenges TNB people face in the United States, Canada, and around the world, more research of this kind is much needed.

Over the last decade, Zachary DuBois and Jae Puckett from Michigan University have co‐led the Trans Resilience and Health in Sociopolitical Contexts study to address exactly this question over time. The project included the states of Oregon, Michigan, Nebraska, and Tennessee with each team measuring in‐depth interview, surveys, and biomarkers at baseline (April 2020) and 1 year later (March 2021). Data collection occurred before and after the election of Joe Biden as well as during the Covid‐19 pandemic. Leading up to the presidential election, anxiety and depression increased while coping and resilience declined (DuBois et al. 2024). But in November 2020, anxiety and depression decreased, and coping improved. However, long‐term trajectories in mental health remained stable (i.e., did not significantly go up or down), with individual factors like perceived social support and access to resources positively influencing mental health outcomes. These patterns highlight the complex effects of sociopolitical events on TNB well‐being. Taken together, peoples' perceptions of their sociopolitical context influenced mental health, minority stress, and identity pride were also ascertained across four of these U.S. states. In one analysis, participants who perceived more negative local attitudes toward TNB people reported higher discrimination, internalized stigma, and anxiety, with these effects partially mediated by minority stressors (Puckett et al. 2022).

**FIGURE 11:**
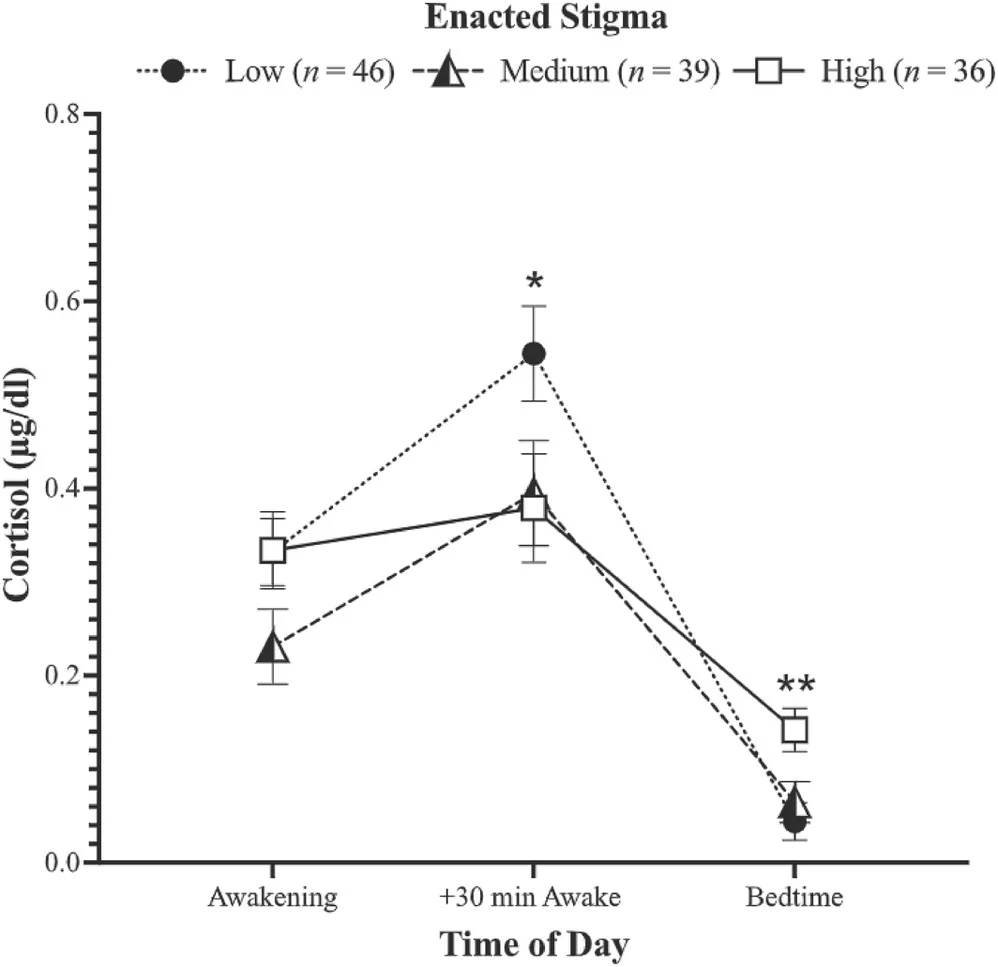
Diurnal cortisol variation among transgender and nonbinary Americans as a function of enacted stigma in the Trans Resilience and Health in Sociopolitical Contexts study. *Source:* DuBois et al. (2024).

In a comprehensive analysis inclusive of transmasculine, transfeminine, and nonbinary people, diurnal cortisol was assessed in association with subscales from the Gender Minority Stress and Resilience Scale (Testa et al. 2022). High enacted stigma was associated with a blunted cortisol awakening response and elevated bedtime cortisol (Figure 11), while community connectedness correlated with healthier stress regulation marked by a more robust cortisol awakening response (DuBois et al. 2017). Interestingly, legal gender affirmation (name and/or gender marker changes) was associated with better psychological and physical health outcomes for TNB participants, partly by reducing exposure to enacted stigma (Puckett et al. 2024). In summary, these findings highlight the need for policies that improve access to legal gender affirmation to support well‐being while also considering both gender minority stress as well as resilience pathways.

## 9 An Intersectional Framework for Minority Stress Biology

**FIGURE 12:**
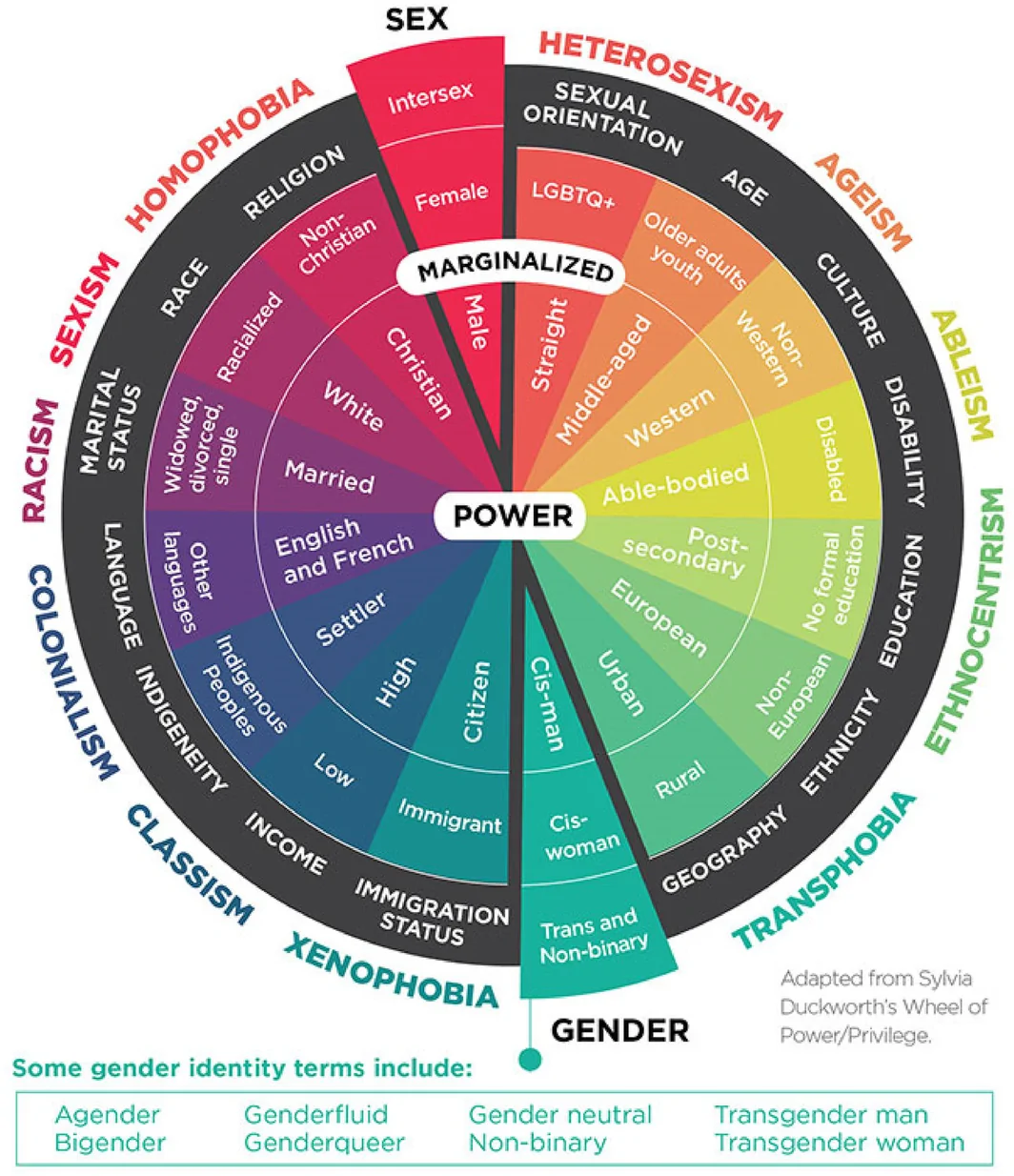
Representation of intersectionality. The example highlighted here illustrates multiple forms of marginalization that can be experienced when focusing on the intersections of sex and gender, not to mention those compounded by diversity in gender identity and/or sexual orientation and many other lived experiences of marginalization. *Source:* Adapted from Sylvia Duckworth, Wheel of Power/Privilege is licensed under Attribution 4.0 International. Reproduced with permission from the Canadian Institutes of Health Research Institute of Gender and Health.

Sexual orientation and gender identity represent central axes of minority stress, and yet they do not operate in isolation. Figure 12 adapted by the Canadian Institutes of Health Research from the Wheel of Power and Privilege underscores that SGM health is fundamentally shaped by intersecting systems of advantage and disadvantage spanning race, ethnicity, socioeconomic status, immigration status, ability, age, and other social positions. Rooted in the foundational work of Crenshaw (1989, 1993), an intersectional framework recognizes that these identities are not additive, but mutually constitutive, producing qualitatively distinct experiences of stigma, access to resources, and health outcomes (Cho et al. 2013).

From this perspective, minority stress must be understood as embodied within broader systems of power, including racism, colonialism, ableism, and heterosexism, which structure both exposure to adversity and opportunities for resilience (Dubois, Puckett, et al. 2022; DuBois, SturtzSreetharan, et al. 2022; Jolly et al. 2025). For example, SGD people who are also racialized, immigrant, or living with disabilities may experience compounded forms of marginalization that amplify stress‐related physiological burden, while also navigating distinct cultural and community‐based sources of resilience. This aligns with emerging population health research demonstrating that structural stigma and social inequities exert measurable effects on biological systems, including neuroendocrine, inflammatory, and epigenetic pathways (Diamond et al. 2021; Geronimus et al. 2006; Hatzenbuehler and McLaughlin 2013; McEwen 2017, 2020).

Integrating intersectionality into the AL framework therefore requires moving beyond uniform assumptions of stress exposure and toward models that account for complexity and heterogeneity in lived experience (Desjardins et al. 2025, 2021; DuBois and Juster 2022). The accumulation of physiological dysregulation captured by AL may differ not only in magnitude but also in patterning or “weathering” (Geronimus et al. 2006) across intersecting identities and contexts. Consistent with critiques of static and individual‐level models, intersectionality highlights that resilience cannot be reduced to personal coping but must be situated within differential access to social, economic, and political resources (Ungar 2011; Ungar et al. 2013). As will be elaborated further on, protective processes such as chosen family, community belonging, and cultural continuity may be particularly salient for multiply marginalized SGM individuals, functioning as critical sites of resistance, adaptation, and physiological recalibration.

An intersectional lens also reinforces the need to conceptualize stress physiology as dynamic and context‐dependent. As sociopolitical environments shift ridiculously rapidly, so too do the configurations of risk and protection that shape biological embedding and embodiment. This is especially relevant in the current era of (de)evolving policies and public attitudes toward SGD populations, where gains in recognition and representation may coexist with persistent or even intensifying forms of structural inequity. The generalized unsafety framework advanced further suggests that these layered and often unpredictable conditions may sustain chronic physiological vigilance, particularly among individuals navigating multiple marginalized identities.

In collaborative work led by Tonia Poteat at Duke University, we are also exploring intersectional stigma in relation to AL as part of the LITE Plus Study. In a study of Black and Latina transgender women living with HIV (Rich et al. 2020), gender affirming hormone therapy duration was initially associated with higher cardiovascular risk; however, only age and AL remained significant predictors after adjusting for sociodemographic confounders (Poteat et al. 2025). In the same sample, the COVID‐19 pandemic was associated with increased chronic stress biomarkers post‐pandemic onset, high AL, and significant COVID‐19 burden, despite high vaccination rates and prevention efforts (Rich et al. 2025). The important work highlights unique vulnerabilities to biopsychosocial stress and the need for greater support during public health crises.

**FIGURE 13:**
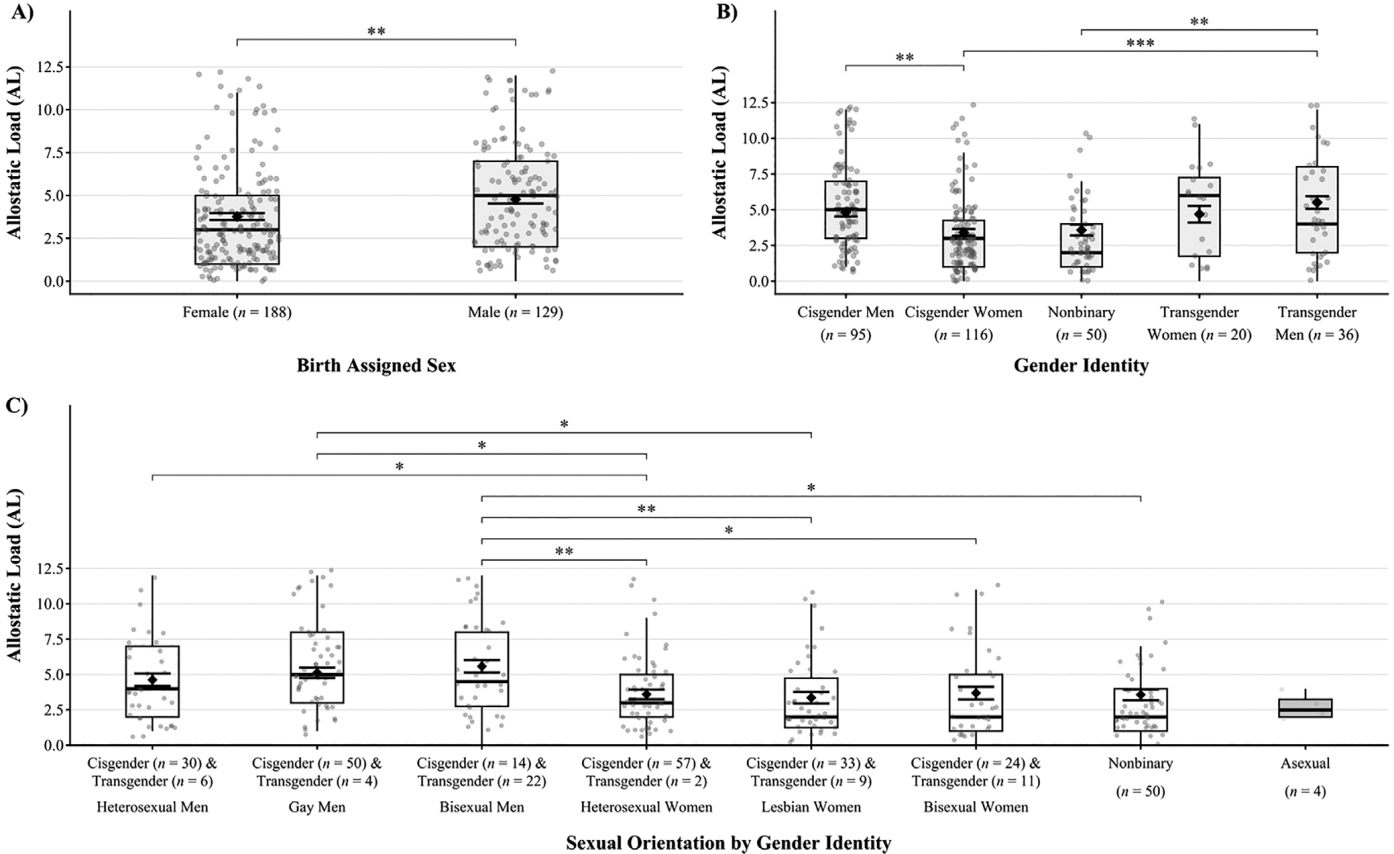
Allostatic load (AL) as a function of: (A) birth‐assigned sex where males show higher AL than females; (B) gender identity where cisgender men and transgender men show the highest AL; and (C) sexual orientation by gender identity where cisgender and transgender gay and bisexual show the highest AL. Note that nonbinary participants show the lowest AL of all sub‐groups. Asexual participants are presented descriptively. Relative to earlier AL studies, gay men no longer are the group with the lowest AL in STARS when an average age of 35. This is a first publication of the Stress and Resilience Study (STARS) based on 16 biomarkers. *Source:* Chuntova et al. (2026).

From 2020 to 2024, my CESAR laboratory held a Canadian Institutes of Health Research *Sex & Gender Science Chair on LGBTQI2S+ Wellness & Resilience*. This Science Chair funded a pioneering research program on stigma, stress physiology, well‐being, cognition, and resilience of SGD people across adulthood. Central to this Chair was the creation of the *Stress and Resilience Study* (STARS) that aims to better understand how stigma shapes biopsychosocial stress and health disparities among SGD adults. In successfully recruiting STARS participants (*N* = 405), we created and curated a unique biobank of saliva, blood, and stool samples for future projects. First findings (Figure 13) show that beyond binary sex (A), gender identity (B) and gender identity by sexual orientation (C) are related to distinct AL patterns (Chuntova et al. 2026). Specifically, *masculine‐spectrum* people & *sexual minority men* show the highest AL levels.

Compared to our earlier AL studies cited earlier on, this indicates that sexual minority men and transgender men appear to “catch up” in their elevated AL in their 30s, suggesting an accelerated aging pattern. In addition, both major and daily intersectional discrimination are associated with elevated AL for the entire STARS sample independent of health‐related behaviors (Chuntova et al. 2026). This shows the direct biological toll of stigma and vulnerability of men to AL. These timely findings highlight structural and social determinants as key drivers of physiological dysregulation in SGM communities. Moving forward, future research must extend this work to older adults who face compounded stigma and remain largely absent from AL studies of SGM populations. This endeavor will inform strategies that can promote SGM resilience and health equity across young to old adulthood.

In summary, situating SGD people's stress physiology within an intersectional framework advances a more precise and equitable science of health disparities. It compels us to examine not only how stress “gets under the skin and skull,” but for whom, under what conditions, and through which configurations of power and privilege. For my research program, this means explicitly integrating intersectional measurement, stratified analyses, and community‐engaged approaches to better capture the complexity of lived experiences. By doing so, we can move toward a next generation of research that more fully reflects diverse populations and more effectively informs interventions aimed at reducing health inequities across multiple dimensions of intersecting identity.

In summary, this body of work reflects a sustained effort in my research program to move beyond binary conceptions of sex and gender in the study of stress physiology. Where early AL research treated sex as a fixed, dichotomous covariate, STARS and related projects instead ask how gender identity, sexual orientation, and their intersections pattern physiological dysregulation in ways a binary model cannot capture. Yet even as this work pushes toward continuums and intersections, I remain conscious that the conceptual contagion of binary thinking: the tendency for statistical and theoretical models, by their very structure, to smuggle binary logic back in, whether through dichotomized biomarkers, categorical predictors, or the analytic convenience of collapsing gender diverse participants into “male/female” or “cisgender/transgender” contrasts for the sake of statistical power. This contagion is subtle specifically because it operates at the level of method rather than intent, and confronting it requires more than adding categories. In continuous collaboration with communities, this refinement requires rethinking how we operationalize sex and gender as constructs in the first place. My hope is that this program of research, and the STARS biobank it has generated, offers not just new empirical findings but a template for resisting this contagion. Sex and gender diversity are phenomena to be modeled in their full complexity rather than identities to be sorted statistically.

## 10 Consideration of Resilience Pathways

The life trajectories that render us vulnerable toward or resilient against diseases of the mind and body are major themes in stress theory, research, and practice (Juster, Seeman, et al. 2016). Akin to the concept of allostasis, resilience in stress circles is defined as a dynamic process that promotes positive adaptation among individuals exposed to severe forms of adversity, stress, and/or trauma (Cicchetti and Garmezy 1993; Luthar et al. 2000; Masten et al. 1990; Rutter 2012). As science furthers our understanding of the multi‐causal mechanisms involved, stress processes and resilience pathways are increasingly conceptualized not as static states, but rather as probabilistic processes that vary according to the contexts and the consequences investigated (Cicchetti and Toth 2009; Rutter 1985; Rutter and Sroufe 2000). This perspective is refreshing in comparison to “doom and gloom” views focused almost entirely on vulnerability or risk factors that dominate the stress literature. This is especially the case for the literature based on sexual and gender minority stress. And yet, the SGD community is strongly requesting that research focus more on positive health and well‐being, a sentiment shared as well by some SGD researchers like me who wish to understand not just how we survive but also how we can thrive and flourish as individuals and communities.

Allostatic mechanisms like the stress response are diverse and span the spectrum of adaptive and maladaptive response patterns. In considering stress and resilience at a biological level—or what is referred to hereon as *biological resilience*—I wish to first define three key interconnected concepts outlined by Karatsoreos and McEwen (2011) regarding: (1) resilience, (2) resistance, and (3) recovery. First and consistent with previous sections, *resilience* is defined as an organism's ability to “rebound” from adversity when one's ability to physiologically function has been tampered with in some negative way; however, the organism is able to adapt by activating allostatic mechanisms that can promote resistance. Second, *resistance* is defined as an organism's ability to withstand adversity and face future stressors with little or no stress response akin to vaccination or stress inoculation that helps bolster immunity. Third, *recovery* is defined as an organism's ability to shut‐down the stress response and other related biological activities back to baseline levels (Karatsoreos and McEwen 2011). All three of these constructs are related to adaptation at a biological level and are central to our understanding biological processes that promote a wide spectrum of outcomes. While the AL framework is embedded within such processes, we next present diverse research areas to provide food for thought to help guide future research in this exciting area.

Stress physiology must be understood in relation to minority stress processes that are inherently dynamic, relational, and structurally embedded. Stigma‐related stressors (e.g., discrimination, vigilance, internalized stigma) are not discrete exposures but ongoing conditions that fluctuate across contexts and over time (Meyer 1995, 2003). As stated earlier, these stress processes are best conceptualized as probabilistic and context‐sensitive, shaped by shifting social environments and access to affirming resources (Cicchetti 2008, 2010). Moreover, while resilience is often operationalized at the individual level, such a focus risks obscuring the central role of community‐based resources, collective identities, and shared infrastructures that scaffold coping under conditions of structural stigma.

## 11 Crisis Competence and Community Resilience

Conceptualizing resilience along a continuum spanning from individuals to communities therefore strengthens our understanding of how differential access to social capital and collective support shapes health inequities across 2S/LGBTQIA+ populations (Meyer 2015). This is particularly salient in the current sociopolitical moment, where rapid changes in SGM rights and visibility may simultaneously generate new opportunities for support and novel forms of stress exposure. In line with critiques of individual‐centric resilience frameworks (Luthar 2003; Ungar 2011; Ungar et al. 2013), resilience among SGM individuals is increasingly understood as a multilevel process that extends beyond coping strategies to include relational (e.g., partner support, chosen family), community (e.g., identity affirmation, collective belonging), and structural (e.g., legal protections, access to care) resources (Meyer 2015).

A fascinating form of community resilience is captured by the crisis competence concept. *Crisis competence* (Friend 1980; Morrow 2001) refers to the process whereby marginalized groups can develop resilience in spite of crisis. Crisis competence is thought to be driven by combinations of risk and resilience factors as individuals develop coping skills, seek social supports, and garner community resources in the face of adversity (Balsam and D'Augelli 2006). Being part of a marginalized group is often associated with stigma; however, it can also help increase skills (Friend 1991), wellness (Kimmel et al. 2006), and even buffer against later crises (Kimmel 1978).

Stressful processes like sexual and gender identity development can be experienced as major life stressors. Notwithstanding, navigation of this proximal stress process can lead to a sense of competence as SGD people develop coping skills across the lifespan. These coping skills act to buffer against experiences of adversity in later life (Berger 1980, 1982; Friend 1980, 1991; Kimmel 1978). Historically, the crisis competence concept focused mainly on later‐life crises when aging. Many older SGMs are already accustomed to independence (e.g., often due to a loss of family support when “coming out”), overcoming diverse forms of stigma, & better adjustment to ageism in general (Friend 1991). The crisis competence concept is of particular interest in studying resilience of those who survived the HIV/AIDS pandemic (Porter et al. 2019). These resilience processes may involve biological mechanisms that have not been explored to date among SGD people.

A central component that promotes crisis competence is social support. In particular, the concept of “*chosen family*” (Dewaele et al. 2011) represents the selection of 2S/LGBTQI+ peers, allies, & networks (Francher and Henkin 1973; Lyons et al. 2012; Quam and Whitford 1992; Sharp 1997) that foster community support. This was explored in the context of the Coronavirus pandemic and ongoing research by my research group. Within the first month after the first detected Covid‐19 infections in Quebec and for the next 100 days thereafter, an online cross‐sectional survey was deployed by Lupien's research group. Across the board, 2S/LGBTQIA+ Quebecers reported significantly higher levels of perceived stress, depression, anxiety, and loneliness compared to cisgender heterosexuals. Those reporting the poorest mental health were gender diverse, bisexual/pansexual, and asexual people, a high‐risk pattern consistent with pre‐pandemic literature (IOM 2011; NIH 2016).

Interestingly, perceived *social support* moderated the relationship between perceived distress and depression for both 2S/LGBTQIA+ and cisgender heterosexuals. Remarkably, this social support buffering effect on distress and depression was four times stronger for 2S/LGBTQIA+ Quebecers (Jacmin‐Park et al. 2022). These early findings strongly encouraged us to further explore other psychosocial factors beyond social support that promote risk or resilience of 2S/LGBTQIA+ Canadians. The crisis competence context has many other potential applications in the current geo‐political context. Further mixed methods and transdisciplinary approaches by our group will further explore stress mechanisms and resilience pathways informed by the crisis competence concept among SGD communities.

For our CESAR laboratory's current and future research directions, this perspective is central. Crisis competence can be operationalized through a biobehavioral lens that examines how these dynamic stress and resilience processes become biologically embodied across interacting systems of sociopolitical oppression and civil resistance. AL provides a valuable, though incomplete, index of cumulative biological risk. When integrated with dynamic frameworks, such as the generalized unsafety theory of stress, advancement in the AL model can be reconceptualized as reflecting not only the “wear and tear” of repeated stress exposure but also the physiological imprint of sustained unsafety and constrained opportunities for recovery.

Importantly, this integrated approach allows for a more nuanced understanding of resilience in SGD communities over time as entangled with minority stress processes. Like allostasis and stress processes, resilience is not a static buffer against stress, but is an emergent, context‐dependent process that can promote both attenuation and repair of physiological dysregulation (Juster et al. 2011; Juster, Russell, et al. 2016; Juster, Seeman, et al. 2016). Constructs such as crisis competence and chosen family are therefore reframed as dynamic, co‐constructed processes that may recalibrate stress physiology over time, particularly in environments where safety, recognition, and belonging are actively cultivated (e.g., GUTS model). This perspective ultimately advances a more precise and socioecological account of sexual and gender minority stress physiology, one that is better aligned with both contemporary resilience science and the lived realities of diverse communities.

In summary, this reconceptualization of resilience demands the same reckoning as our retreat from rigid, binary sex and gender categories. Both require resisting the conceptual contagion of binary thinking that saturates stress science, whether coded as vulnerable‐versus‐resilient, static‐versus‐shifting, or male‐versus‐female. Just as gender resists the flattening force of our statistical models, resilience resists reduction to a trait one possesses or lacks. Indeed, resilience is probabilistic, patterned, and profoundly context‐dependent, unfolding differently across intersecting identities, communities, and historical moments. Crisis competence, chosen family, and generalized unsafety are not oppositional poles to AL's “wear and tear”, but entangled, co‐constructed processes that recalibrate one another over time. Precision, not polarity, is what this science demands. I strongly believe that only by resisting binary categories of harm and healing can AL research reflect the full complexity of the populations it claims to serve. In a truly transdisciplinary spirit, this reflection forces us to move from categories toward continuums at every level, biological, psychological, and social alike.

## 12 Conceptual Model: Minority Stress, Allostatic Load, and Multidimensional Health

**FIGURE 14:**
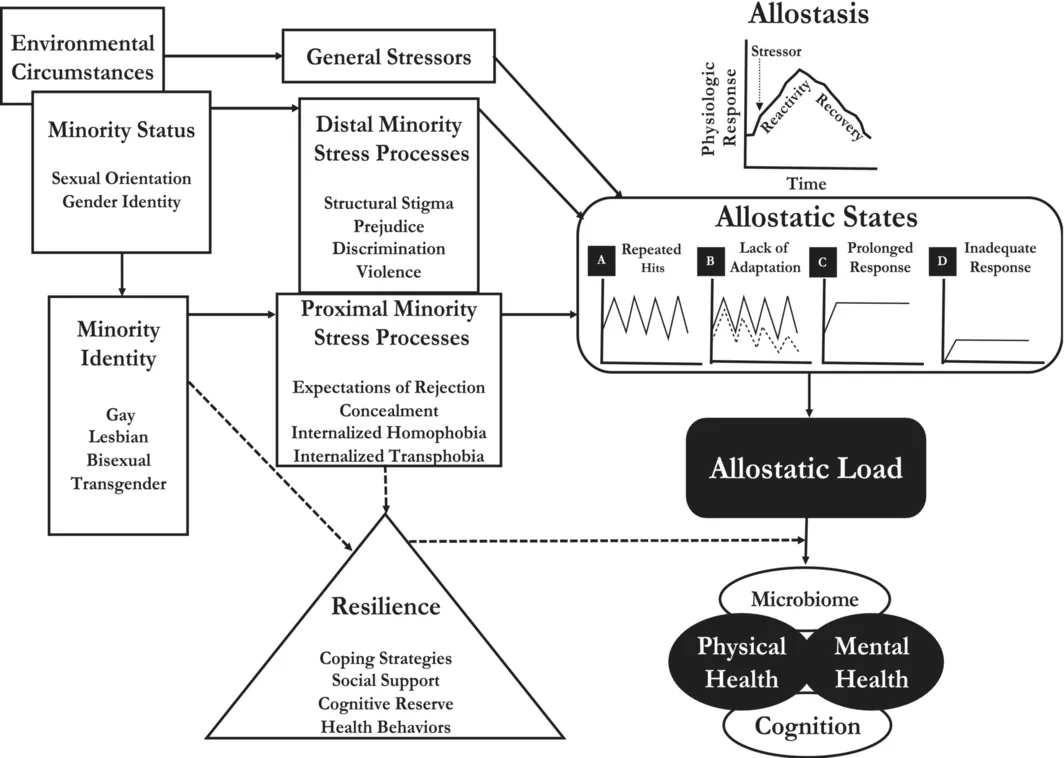
Conceptual model linking sexual and gender minority stress to stress processes, allostatic load, and health. Note that microbiome and cognitive measures are the focus of complementary research programs. *Source:* Adapted from Juster et al. (2017).

The conceptual model presented next situates sexual and gender minority health within an integrative, biobehavioral framework linking minority stress processes to AL and downstream health outcomes across multiple physiological systems. Building on minority stress presented to the left (Figure 14), SGD people are disproportionately exposed to chronic and socially patterned stressors, including intersectional stigma, discrimination, and identity‐based marginalization that operate across individual, interpersonal, and institutional levels. In this model, minority status related to sexual orientation and/or gender identity gives rise to lived minority identities (e.g., gay, lesbian, bisexual, transgender, nonbinary), which are embedded within dynamic sociocultural contexts that shape both stress exposure and access to protective resources.

At the physiological level presented to the right (Figure 14), these stress exposures are hypothesized to influence patterns of allostatic regulation across time. Consistent with the AL framework, repeated or chronic activation of stress‐responsive systems can give rise to distinct allostatic states. As illustrated in the model, these include (A) repeated “hits,” (B) inefficient adaptation, (C) prolonged activation, and (D) inadequate responses, each of which reflects dysregulation in the timing, magnitude, or recovery of physiological stress responses. Over time, the accumulation of such dysregulation is indexed as AL representing the multisystem biological embodiment of lived experience.

A central innovation of this conceptual model is the explicit integration of emerging biological pathways that may mediate and/or moderate the associations among AL and wide spectrum of health outcomes. In particular, I emphasize the role of the gut microbiome as a dynamic interface between social experience and host physiology, with growing evidence linking stress exposure to microbial composition, inflammation, and neurobehavioral functioning (Juster et al. 2020). Similarly, processes of “inflamm‐aging” refer to chronic, low‐grade systemic inflammation (Franceschi et al. 2000) that are conceptualized as downstream consequences of sustained physiological activation, with implications for accelerated aging and chronic disease risk. As detailed in a scoping review (Christian et al. 2021), the *Biopsychosocial Minority Stress Framework* aims to explain how stigma‐related stress influence immune function, inflammation, and long‐term health. Additional mechanisms, including neurotrophin signaling (e.g., brain‐derived neurotrophic factor), epigenetic modifications, and other molecular and sub‐cellular pathways (Picard et al. 2014, 2017) are all emerging areas of our future research directions.

Importantly, this conceptual model also foregrounds resilience as a multilevel, dynamic process that can buffer, recalibrate, and potentially reverse stress‐related dysregulation. Moving beyond individual‐centric notions of coping, resilience is conceptualized as arising from interactions among relational (e.g., intimate partner support), community (e.g., chosen family, identity affirmation), and structural (e.g., policies, access to affirming care) resources. Within SGD populations, constructs such as crisis competence and chosen family are therefore understood as emergent, co‐regulated processes that may shape both exposure to stress and the efficiency of physiological recovery.

Guiding my research program and that of the CESAR team, this framework supports a multimethod approach that integrates experimental paradigms (e.g., Trier Social Stress Test), longitudinal cohort data, and biomarker‐based assessments to examine how minority stress becomes biologically embodied. Critically, how do resilience processes mitigate or reverse these effects? I believe many answers are possible by linking social conditions to multisystem physiology and diverse health outcomes that include mental, physical, and cognitive functioning. In summary, this conceptual model aims to advance a more precise, socioecological valid account of SGD‐related health disparities and to inform interventions that target not only risk, but also resistance, recovery, and resilience across levels of analysis.

## 13 Conclusions

In closing, 2S/LGBTQIA+ people continue to face discrimination simply because of who they are, who they love, and how they express themselves. Beyond the struggles of SGD people, social movements often involve clashes between groups, leading to deep societal tensions between and sometimes within communities. To address these divisions and the inequalities they create, courageous individuals have engaged in activism to push for social and legal change. Academia has long stood in support of progress and fostering safe spaces for SGD people; however, we are living in trying times where our institutions are under political attack. With so much uncertainty regarding SOGI representation in surveys and census, it is imperative to not render the SGD community invisible. As has been shown in this narrative review of my research program over the last 15 years, national datasets are needed to provide the statistical power to analyze AL and health based on sexual orientation and hopefully soon gender identity. This is a pre‐requisite to study how health is shaped by structural stigma in the form of homophobia and transphobia from the micro‐level to the macro‐level.

## Author Contributions

**Robert‐Paul Juster:** conceptualization, data curation, formal analysis, funding acquisition, investigation, methodology, project administration, supervision, writing and editing.

## Funding

R.‐P.J. receives salary support from the Fonds de Recherche du Québec Sante (Jr1–269532: https://doi.org/10.69777/269532 and Jr 2–332273 https://doi.org/10.69777/332273) and Fondation de l'Institut Universitaire en Santé Mentale de Montréal.

## Ethics Statement

The author has nothing to report.

## Conflicts of Interest

The author declares no conflicts of interest.

## Data Availability

The data that support the findings of this study are available on request from the corresponding author. The data are not publicly available due to privacy or ethical restrictions.
